# Event-triggered MPC-PID based trajectory tracking control for differential drive mobile robots

**DOI:** 10.1371/journal.pone.0354699

**Published:** 2026-07-29

**Authors:** Maike Wang, Gang Zhang, Duansong Wang

**Affiliations:** School of Electrical and Photoelectric Engineering, West Anhui University, Lu’an, Anhui, China; Zhejiang University, CHINA

## Abstract

Periodic model predictive control (MPC) for differential-drive mobile robots requires online optimization at every sampling instant, which can be redundant during near-steady tracking. This study develops an event-triggered hierarchical MPC–PID framework, termed ET–MPC–PID, to reduce the outer-loop optimization burden while accounting for actuator-side execution effects. The outer MPC updates the velocity setpoint only when a normalized event condition is satisfied or the maximum holding interval is reached. A fixed-period PID-form velocity servo with conditional integration regulates the actuator channel under lag and saturation. The formulation distinguishes holding-induced decision discrepancy from actuator-side mismatch and provides a local practical boundedness characterization. Simulations on double lane-change and figure-eight trajectories compare periodic PID, periodic MPC, periodic MPC–PID, an external variable-horizon ET–MPC reference, and event-triggered ablations. ET–MPC–PID reduces the QP call rate to 16.9% and 22.3% of periodic MPC while outperforming the no-PID event-triggered variant. Sensitivity, Pareto, runtime, and 100-trial Monte Carlo analyses further demonstrate the accuracy–computation trade-off and empirical robustness under sampled execution uncertainty.

## Introduction

Differential-drive mobile robots are widely used in environmental inspection, warehousing logistics, service robotics, and intelligent manufacturing because of their simple mechanical structure, high maneuverability, and favorable energy efficiency. However, accurate trajectory tracking remains challenging in practical operation because of nonholonomic constraints, actuator bandwidth limitations, wheel–ground interaction changes, payload variations, unmodeled dynamics, and external disturbances. Villalobos-Aranda et al. [1] proposed an almost globally convergent trajectory-tracking controller for differential-drive wheeled mobile robots, improving convergence under large variations in initial conditions. From a sensing and implementation perspective, Yan et al. [2] investigated trajectory tracking using only position and velocity measurements, which is relevant for low-cost mobile platforms with limited sensing capability. These studies indicate that trajectory-tracking performance depends not only on nominal controller design, but also on practical factors such as sensing limitations, actuator response, and real-time computational resources.

Model predictive control (MPC) is attractive for mobile-robot trajectory tracking because it can explicitly handle input constraints, input-rate limits, and multi-objective performance requirements within a receding-horizon optimization framework. Tang et al. [3] introduced geometric MPC to improve tracking while preserving geometric consistency. Zhang et al. [4] incorporated Lyapunov-based input constraints to support feasibility and stability analysis, and Peng et al. [5] combined disturbance observation with predictive control to improve robustness under external disturbances. Despite these advantages, conventional periodic MPC requires solving an online optimization problem at every sampling instant. This may lead to redundant computation when tracking errors and reference variations are small. Moreover, frequent outer-loop updates can generate rapidly varying velocity setpoints for the low-level actuator system, thereby amplifying the influence of actuator lag and causing unnecessary control fluctuations.

To improve real-time implementability, many AGV/AMR systems adopt hierarchical control architectures, in which an outer loop generates motion commands or velocity setpoints and an inner loop performs fast velocity servoing and disturbance rejection. de Omena et al. [6] proposed a two-tier MPC architecture assisted by edge computing to reduce online computation in industrial applications, while Tang et al. [7] studied trajectory tracking under motion/force coordination constraints, reflecting the need for accurate yet smooth execution. More recently, Guo et al. [8] developed an MPC–PID double-loop trajectory-tracking strategy for intelligent sweeping vehicles, where MPC regulates the robot pose and PID adjusts the longitudinal speed. Such hierarchical MPC–PID designs are practically meaningful because the inner-loop servo can compensate part of the mismatch between optimized setpoints and actual actuator responses. However, most existing hierarchical MPC–PID methods retain periodic outer-loop optimization and therefore do not systematically reduce redundant MPC solves during near-steady tracking.

Event-triggered control provides an update-on-demand mechanism for reducing communication and computation while maintaining acceptable closed-loop performance. A recent overview of event-triggered control is provided by Zhang et al. [9]. In the MPC context, Li et al. [10] studied event-based MPC for nonlinear systems with dynamic disturbances to avoid unnecessary optimization, and later proposed disturbance-prediction-based adaptive triggering to improve adaptability [11]. Yang et al. [12] incorporated a disturbance observer into ET-MPC for nonlinear input-affine systems, while Deng et al. [13] and Luo et al. [14] addressed robustness, terminal constraints, trigger-density regulation, and Zeno-like behavior. Gu et al. [15] investigated tube-based event-triggered stochastic MPC under additive uncertainty, and Shen et al. [16] examined resilient nonlinear MPC with a dynamic event-triggered strategy under denial-of-service attacks. Foundational ET-MPC studies further established how intermittent sampling, temporal constraints, and robust feasibility can be analyzed in a principled manner [17–21].

Event-triggered predictive control has also been extended to vehicle and mobile-robot tracking tasks. Zou et al. [22] designed an event-triggered nonlinear MPC scheme for trajectory tracking of unmanned vehicles to improve real-time performance. Zhang et al. [23] proposed an event-triggered model predictive-preview control strategy for autonomous-vehicle trajectory tracking, where the triggering condition was designed by considering stability and feasibility. More recently, Zhang et al. [24] developed an event-triggered MPC approach for autonomous-vehicle trajectory tracking. For mobile robots, Sun et al. [25] developed a disturbance event-triggered predictive tracking controller for a 4WIS–4WID mobile robot to reduce the influence of wheel–ground interaction disturbances.

These studies demonstrate that ET-MPC can reduce the online computational burden of trajectory tracking. Nevertheless, most existing ET-MPC tracking designs focus mainly on the outer-loop predictive controller itself. The coupled effects of event-triggered setpoint holding, actuator lag, input saturation, and fixed-period inner-loop velocity-servo dynamics have received less explicit attention, particularly for differential-drive mobile robots. In addition, direct same-platform comparisons between an external ET-MPC reference and an ET-MPC–PID implementation remain limited. Therefore, the present study includes an external ET-MPC reference baseline following the event-triggered MPC design reported in [24], enabling the comparative simulations to distinguish the effects of the event-update policy from those of fixed-period inner-loop velocity compensation.

Related event-triggered PID and integrated guidance–control studies in autonomous surface vehicles also provide useful reference points for the present work. Zhu et al. [26] proposed an event-triggered adaptive PID fault-tolerant controller for underactuated autonomous surface vehicles under saturation constraints, showing that event-triggered PID-type mechanisms can reduce actuator update effort while maintaining bounded closed-loop signals. Zhu et al. [27] developed an FTILOS-based path-following method for underactuated USVs with obstacle avoidance, using an integrated planning–guidance–control design to address sideslip compensation, dynamic uncertainty, and safe path generation. These studies are relevant because they consider practical issues such as event-triggering, saturation, fault tolerance, obstacle avoidance, and integrated control. However, their control objectives, plant models, and design structures differ from those considered here: they focus mainly on underactuated marine vehicles and adaptive or integrated guidance–control designs, whereas this paper considers differential-drive mobile robots and the interaction between an event-triggered outer-loop MPC and a fixed-period inner-loop PID velocity servo.

Table 1 summarizes the differences between representative periodic, hierarchical, and event-triggered trajectory-tracking methods, including the external ET-MPC reference adopted in the comparative simulations. Existing periodic MPC methods provide constrained optimization but do not reduce the outer-loop update frequency. Existing MPC–PID methods improve low-level execution but usually retain periodic optimization. Existing ET-MPC methods reduce online computation, but they generally do not explicitly examine the influence of setpoint holding on a fixed-period inner-loop velocity servo with actuator dynamics. This motivates the present event-triggered hierarchical MPC–PID design.

**Table 1:** Comparison with representative periodic, hierarchical, and event-triggered trajectory-tracking methods.

Work	Platform	ET	Hier.	Actuator / servo	Stability / robustness	Main gap relative to this work
Tang et al. [3]	Mobile robot	No	No	Not focused	Geometric tracking	No event-triggered outer loop; no PID velocity servo.
de Omena et al. [6]	AGV / AMR	No	Yes	Partly addressed	Real-time implementation	No event-triggered outer-loop MPC–PID design.
Guo et al. [8]	Sweeping vehicle	No	Yes	PID speed regulation	Simulation verification	Periodic MPC–PID; no event-triggered optimization.
Zou et al. [22]	Unmanned vehicle	Yes	No	No explicit low-level velocity servo	Feasibility / stability	No analysis of ET holding with a fixed-period PID velocity servo.
Zhang et al. [24]	Autonomous vehicle	Yes	No	No explicit low-level velocity servo	Trajectory-tracking validation	External ET-MPC reference; no differential-drive actuator-chain treatment or inner-loop PID servo.
Sun et al. [25]	4WIS–4WID robot	Yes	Not emphasized	Disturbance considered	Wheel–ground robustness	Not an ET-MPC plus fixed-period PID velocity-servo structure.
Zhu et al. [26]	Underactuated ASV	Yes	PID-type adaptive	Saturation / faults	Closed-loop boundedness	Marine ET adaptive PID; no constrained MPC outer loop.
Zhu et al. [27]	Underactuated USV	No	Integrated guidance–control	No PID velocity servo	Uniform boundedness	USV path following; not ET-MPC–PID for differential-drive robots.
**This work**	**Differential-drive robot**	**Yes**	**Yes**	**Actuator dynamics, saturation, PID servo**	**Local practical boundedness under bounded mismatch**	**Outer-loop ET-MPC, fixed-period PID velocity servo, actuator-mismatch-aware analysis, same-platform external ET-MPC comparison, and robustness evaluation.**

ET: event-triggered; Hier.: hierarchical; ASV: autonomous surface vehicle; USV: unmanned surface vehicle.

This paper addresses the above practical gap by developing an event-triggered hierarchical MPC–PID trajectory-tracking framework for differential-drive mobile robots. The hierarchical MPC–PID architecture itself is not claimed as a stand-alone novelty. Instead, the contribution lies in integrating an event-triggered outer-loop constrained MPC with a fixed-period inner-loop PID velocity servo and in explicitly evaluating the resulting effects of setpoint holding, actuator lag, input saturation, and execution mismatch.

The research question is whether the online optimization frequency of constrained MPC can be reduced while retaining practically acceptable tracking performance when the low-level actuator response is explicitly considered. To address this question, the outer loop solves an error-augmented receding-horizon MPC problem only when the triggering condition is satisfied or when the maximum holding time is reached. The inner loop tracks the resulting velocity setpoint at a fixed sampling period, while first-order actuator dynamics and saturation represent the physical execution chain. The resulting closed-loop behavior is assessed through a same-platform external ET-MPC comparison, ablation studies, trigger-parameter sensitivity and Pareto analyses, and Monte Carlo robustness tests under execution uncertainty.

The main contributions of this study are summarized as follows.

1. An event-triggered hierarchical MPC–PID trajectory-tracking scheme is developed for differential-drive mobile robots. The outer-loop constrained MPC is updated on demand, whereas a fixed-period inner-loop PID velocity servo regulates the actuator response between consecutive MPC updates. This structure explicitly distinguishes the MPC setpoint, saturated actuator command, and actual actuator velocity.
2. The influence of event-triggered setpoint holding, actuator lag, and input saturation is represented as a bounded execution mismatch. Under a local feasible operating region and bounded mismatch assumptions, a local practical boundedness characterization is derived to explain how the triggering threshold, maximum holding time, and inner-loop response affect the attainable tracking neighborhood.
3. A comprehensive simulation study is conducted on double lane-change and figure-eight trajectories. The evaluation includes periodic PID, periodic MPC, periodic MPC–PID, an external ET-MPC reference following [24], and internal event-triggered ablation variants. A two-parameter sensitivity and Pareto analysis is conducted for both trajectories, and 100 Monte Carlo trials per scenario are conducted to evaluate robustness under execution uncertainty.

The remainder of this paper is organized as follows. Section 2 formulates the differential-drive tracking problem and derives the error-augmented discrete model. Section 3 presents the proposed ET-MPC–PID framework, including the outer-loop MPC, event-triggering mechanism, inner-loop PID velocity servo, and local practical boundedness analysis. Section 4 reports the same-platform controller comparison, ablation study, event-update behavior, trigger-parameter sensitivity and Pareto analyses, and Monte Carlo robustness evaluation. Section 5 concludes the paper and discusses future research directions.

## Problem formulation and modeling of differential-drive mobile robots

### Kinematic modeling of differential-drive mobile robots

**Fig 1:**
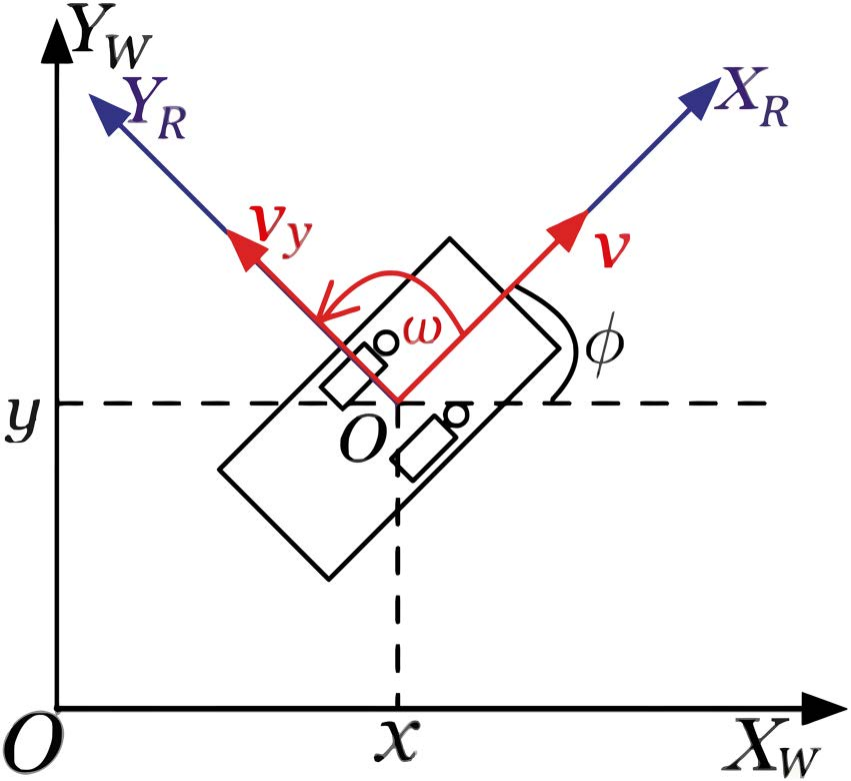
Kinematic model of the robot in the world coordinate frame.

To achieve pose tracking of wheeled differential-drive mobile robots, a standard nonholonomic kinematic model is used to describe the robot planar pose in a world coordinate system. This model consists of three states and two velocity inputs derived from geometric kinematic relationships. Fig. 1 illustrates the motion decomposition, while Table 2 summarizes the key symbols used in the kinematic model.

**Table 2:** Symbol definitions for the robot kinematic model.

Symbol	Description
$X_{w}\;-\;O\;-\;Y_{w}$	World inertial planar frame
$X_{R}\;-\;O\;-\;Y_{R}$	Robot body-fixed frame
*x*	COM *x*-position in the world frame
*y*	COM *y*-position in the world frame
ϕ	Heading angle of the robot body with respect to $X_{w}$
*v*	Linear velocity along the robot longitudinal axis
ω	Yaw rate about the out-of-plane axis

Let the robot pose be represented by


$$\begin{array}{c} 𝐱={\left[\begin{array}{ccc} x & y & \phi \end{array}\right]}^{T}, \end{array}$$ (1)

where *x* and *y* are the coordinates of the robot center of mass in the world frame $X_{w}\;-\;O\;-\;Y_{w}$, and ϕ is the heading angle measured counterclockwise from the $X_{w}$ axis. The differential-drive platform satisfies the nonholonomic constraint that the lateral velocity in the body-fixed frame is zero.

In an ideal kinematic model, the velocity input is usually written as $𝐮=[v,\omega {]}^{T}$. In the proposed hierarchical controller, however, the velocity generated by the outer-loop MPC is not necessarily equal to the velocity actually applied to the robot because of event-triggered input holding, inner-loop velocity servoing, input saturation, and actuator dynamics. Therefore, the actual velocity applied to the robot is denoted by


$$\begin{array}{c} {𝐮}_{act}={\left[\begin{array}{cc} v_{act} & \omega_{act} \end{array}\right]}^{T}. \end{array}$$ (2)

Using a planar rotation by ϕ to transform the body-frame velocity into the inertial frame, the continuous-time kinematic model driven by the actual actuator velocity is


$$\begin{array}{cc} \dot{x} & =v_{act}\cos\phi ,\\ \dot{y} & =v_{act}\sin\phi ,\\ \dot{\phi } & =\omega_{act}. \end{array}$$ (3)

Eq (3) describes the continuous-time kinematic model of the differential-drive mobile robot in the world frame. Its compact matrix form is


$$\begin{array}{c} \dot{𝐱}=[\begin{array}{cc} \cos\phi & 0\\ \sin\phi & 0\\ 0 & 1 \end{array}]{𝐮}_{act}. \end{array}$$ (4)

Eqs (3)–(4) provide the basis for the subsequent prediction model and controller design. The key point is that the physical plant is driven by ${𝐮}_{act}$, whereas the MPC optimizes a nominal velocity setpoint. This distinction is retained in the following modeling process to explicitly represent actuator mismatch.

The modeling assumptions used in this study are summarized as follows. First, lateral wheel–ground slip is neglected, and the differential-drive robot is assumed to satisfy the nonholonomic rolling constraint. Second, the outer-loop MPC is designed based on the kinematic model and the local error-augmented linearized prediction model. Third, actuator delay, saturation, and unmodeled fast dynamics are represented by a bounded mismatch between the MPC velocity setpoint and the actual actuator velocity. This mismatch is further regulated by the inner-loop PID velocity servo introduced in the controller design section.

### Input signal chain and actuator model

To clarify the hierarchical control architecture, three velocity signals are distinguished in this study.

First, the outer-loop MPC generates the velocity setpoint


$$\begin{array}{c} {𝐮}_{set,k}={\left[\begin{array}{cc} v_{set,k} & \omega_{set,k} \end{array}\right]}^{T}. \end{array}$$ (5)

This signal is the nominal control input optimized by the MPC. In the event-triggered scheme, ${𝐮}_{set,k}$ is updated only when the triggering condition is satisfied or when the maximum holding time is reached. Otherwise, the previously generated setpoint is held.

Second, the actuator command after the inner-loop controller and saturation is denoted as


$$\begin{array}{c} {𝐮}_{cmd,k}={\left[\begin{array}{cc} v_{cmd,k} & \omega_{cmd,k} \end{array}\right]}^{T}. \end{array}$$ (6)

For the proposed ET-MPC-PID controller, ${𝐮}_{cmd,k}$ is generated by the PID velocity servo and then limited by actuator saturation. For the ET-MPC ablation controller without the PID inner loop, the command input is obtained by directly saturating the MPC setpoint.

Third, the actual velocity applied to the robot is


$$\begin{array}{c} {𝐮}_{act,k}={\left[\begin{array}{cc} v_{act,k} & \omega_{act,k} \end{array}\right]}^{T}. \end{array}$$ (7)

This actual velocity drives the plant model in Eq (3). In general,


$$\begin{array}{c} {𝐮}_{set,k}\text{≠}{𝐮}_{cmd,k}\text{≠}{𝐮}_{act,k}, \end{array}$$ (8)

because the signal chain includes event-triggered setpoint holding, inner-loop servoing, actuator saturation, and first-order actuator dynamics.

The admissible MPC velocity-setpoint bounds are expressed as


$$\begin{array}{c} {𝐮}_{set,min}\leq {𝐮}_{set,k}\leq {𝐮}_{set,max}, \end{array}$$ (9)

where


$$\begin{array}{c} {𝐮}_{set,min}={\left[\begin{array}{cc} v_{set,min} & \omega_{set,min} \end{array}\right]}^{T},\;{𝐮}_{set,max}={\left[\begin{array}{cc} v_{set,max} & \omega_{set,max} \end{array}\right]}^{T}. \end{array}$$ (10)

The input-rate constraint used in the MPC is written as


$$\begin{array}{c} \Delta {𝐮}_{set,min}\leq \Delta {𝐮}_{set,k}\leq \Delta {𝐮}_{set,max}, \end{array}$$ (11)

where


$$\begin{array}{c} \Delta {𝐮}_{set,k}={𝐮}_{set,k}-{𝐮}_{set,k-1}. \end{array}$$ (12)

The actuator command is subject to component-wise physical saturation:


$$\begin{array}{c} {𝐮}_{cmd,k}={sat}_{{𝒰}_{cmd}}\;\left({𝐮}_{raw,k}\right), \end{array}$$ (13)

where ${𝐮}_{raw,k}$ denotes the unsaturated output of the inner-loop velocity servo and


$$\begin{array}{c} {𝒰}_{cmd}=\left\{𝐮:{𝐮}_{cmd,min}\leq 𝐮\leq {𝐮}_{cmd,max}\right\}. \end{array}$$ (14)

The saturation operator is defined as


$$\begin{array}{c} {sat}_{{𝒰}_{cmd}}(𝐮)=[\begin{array}{c} {sat}_{v}(v)\\ {sat}_{\omega }(\omega ) \end{array}], \end{array}$$ (15)

with


$$\begin{array}{c} {sat}_{v}(v)=\min\left(\max(v,v_{cmd,min}),v_{cmd,max}\right),\;{sat}_{\omega }(\omega )=\min\left(\max(\omega ,\omega_{cmd,min}),\omega_{cmd,max}\right). \end{array}$$ (16)

To represent actuator lag and low-level response delay, the actuator dynamics are modeled as a first-order discrete-time system:


$$\begin{array}{c} {𝐮}_{act,k+1}=A_{u}{𝐮}_{act,k}+\left(I-A_{u}\right){𝐮}_{cmd,k}, \end{array}$$ (17)

where


$$\begin{array}{c} A_{u}=[\begin{array}{cc} \alpha_{v} & 0\\ 0 & \alpha_{\omega } \end{array}],\;\alpha_{v}=\exp\left(-\frac{T_{s}}{\tau_{v}}\right),\;\alpha_{\omega }=\exp\left(-\frac{T_{s}}{\tau_{\omega }}\right). \end{array}$$ (18)

Here, $\tau_{v}$ and $\tau_{\omega }$ are the time constants of the linear and angular velocity channels, respectively. A larger time constant indicates a slower actuator response. This model explains why the actual velocity may lag behind the setpoint even when the command input satisfies the physical constraints.

The actuator-side execution mismatch is defined as


$$\begin{array}{c} {𝐝}_{act,k}={𝐮}_{act,k}-{𝐮}_{set,k}. \end{array}$$ (19)

This quantity represents the discrepancy between the held MPC velocity setpoint and the velocity actually applied to the robot. It captures the effects of actuator lag, command saturation, and unmodeled fast execution dynamics for a given setpoint.

The effect of retaining an outdated outer-loop decision is distinguished separately. Let ${𝐮}_{set,k}^{⋆}$ denote the velocity setpoint that would be obtained if the MPC optimization were solved at time *k*. The holding-induced decision discrepancy is defined as


$$\begin{array}{c} {𝐝}_{hold,k}={𝐮}_{set,k}^{⋆}-{𝐮}_{set,k}. \end{array}$$ (20)

Thus, ${𝐝}_{hold,k}$ characterizes the deviation caused by retaining the previously optimized setpoint, whereas ${𝐝}_{act,k}$ characterizes the actuator-side deviation relative to that held setpoint. Their effects are introduced separately in the subsequent local perturbation analysis.

### Model discretization and state-space formulation

To enable online implementation on a digital controller, the continuous-time kinematic model in Eq (3) is discretized with sampling period $T_{s}$. The actual plant model is obtained by forward Euler discretization as


$$\begin{array}{cc} x_{k+1} & =x_{k}+T_{s}v_{act,k}\cos\phi_{k},\\ y_{k+1} & =y_{k}+T_{s}v_{act,k}\sin\phi_{k},\\ \phi_{k+1} & =\phi_{k}+T_{s}\omega_{act,k}. \end{array}$$ (21)

This equation describes the physical robot motion driven by ${𝐮}_{act,k}$. In contrast, the MPC prediction model is constructed with respect to the nominal velocity setpoint ${𝐮}_{set,k}$. This separation allows the controller to optimize a constrained nominal input while the plant model still reflects actuator lag and saturation.

For trajectory tracking, let


$$\begin{array}{c} {𝐱}_{k}^{r}={\left[\begin{array}{ccc} x_{k}^{r} & y_{k}^{r} & \phi_{k}^{r} \end{array}\right]}^{T},\;{𝐮}_{k}^{r}={\left[\begin{array}{cc} v_{k}^{r} & \omega_{k}^{r} \end{array}\right]}^{T} \end{array}$$ (22)

denote the reference pose and reference velocity at time *k*, respectively. The world-frame pose tracking error is defined as


$$\begin{array}{c} {\tilde{𝐱}}_{k}^{w}≜{𝐱}_{k}-{𝐱}_{k}^{r}={\left[\begin{array}{ccc} \Delta x_{k} & \Delta y_{k} & \Delta \phi_{k} \end{array}\right]}^{T}, \end{array}$$ (23)

where


$$\begin{array}{c} \Delta \phi_{k}=wrap\;\left(\phi_{k}-\phi_{k}^{r}\right)\in (-\pi ,\pi ], \end{array}$$ (24)

and wrap(·) maps an angle to (−π,π]. This avoids artificial discontinuities when the heading angle crosses ±π.

To explicitly incorporate input-rate constraints in the MPC formulation, the control increment is selected as the decision variable:


$$\begin{array}{c} \Delta {𝐮}_{k}≜{𝐮}_{set,k}-{𝐮}_{set,k-1}. \end{array}$$ (25)

The previous setpoint ${𝐮}_{set,k-1}$ is appended to the error state to form the augmented state vector


$$\begin{array}{c} \xi_{k}=[\begin{array}{c} {\tilde{𝐱}}_{k}^{w}\\ {𝐮}_{set,k-1} \end{array}]. \end{array}$$ (26)

A first-order Taylor expansion of the nominal discrete-time kinematic model around the reference pair $\left({𝐱}_{k}^{r},{𝐮}_{k}^{r}\right)$, neglecting higher-order terms, yields an error-augmented linear time-varying model:


$$\begin{array}{c} \xi_{k+1}=A_{k}\xi_{k}+B_{k}\Delta {𝐮}_{k},\;y_{k}=C_{k}\xi_{k},\;C_{k}=[\;T(\phi_{k}^{r})\;,\;0\;]. \end{array}$$ (27)

In Eq (27), $y_{k}=C_{k}\xi_{k}$ denotes the MPC prediction output, namely the tracking error expressed in a reference-aligned local frame. The matrix $C_{k}$ is constructed from the rotation matrix $T(\phi_{k}^{r})$, which is defined in Eq (28).

The system matrix $A_{k}$ and input matrix $B_{k}$ are computed from the Jacobians evaluated at the reference pair $\left({𝐱}_{k}^{r},{𝐮}_{k}^{r}\right)$:


$$\begin{array}{c} A_{k}=[\begin{array}{ccccc} 1 & 0 & -T_{s}v_{k}^{r}\sin\phi_{k}^{r} & T_{s}\cos\phi_{k}^{r} & 0\\ 0 & 1 & T_{s}v_{k}^{r}\cos\phi_{k}^{r} & T_{s}\sin\phi_{k}^{r} & 0\\ 0 & 0 & 1 & 0 & T_{s}\\ 0 & 0 & 0 & 1 & 0\\ 0 & 0 & 0 & 0 & 1 \end{array}],\;B_{k}=[\begin{array}{cc} T_{s}\cos\phi_{k}^{r} & 0\\ T_{s}\sin\phi_{k}^{r} & 0\\ 0 & T_{s}\\ 1 & 0\\ 0 & 1 \end{array}]. \end{array}$$

To ensure consistency among prediction, cost evaluation, and event-triggering feedback, the MPC output, the cost-function error, and the triggering error are all defined using the geometric tracking error expressed in a reference-aligned local frame. The corresponding reference-aligned tracking error is


$$\begin{array}{c} e_{k}≜[\begin{array}{c} e_{x,k}\\ e_{y,k}\\ e_{\phi ,k} \end{array}]=\underset{≜\;T(\phi_{k}^{r})}{\underbrace{\left[\begin{array}{ccc} \cos\phi_{k}^{r} & \sin\phi_{k}^{r} & 0\\ -\sin\phi_{k}^{r} & \cos\phi_{k}^{r} & 0\\ 0 & 0 & 1 \end{array}\right]}}{\tilde{𝐱}}_{k}^{w},\;e_{\phi ,k}=\Delta \phi_{k}. \end{array}$$ (28)

With this definition, the MPC output satisfies $y_{k}=e_{k}$.

Because the plant is driven by ${𝐮}_{act,k}$ while the prediction model uses ${𝐮}_{set,k}$, the actual model can be written as the nominal model plus an actuator-side execution mismatch. Substituting


$$\begin{array}{c} {𝐮}_{act,k}={𝐮}_{set,k}+{𝐝}_{act,k} \end{array}$$ (29)

into Eq (21) gives


$$\begin{array}{cc} x_{k+1} & =x_{k}+T_{s}\left(v_{set,k}+d_{v,k}\right)\cos\phi_{k},\\ y_{k+1} & =y_{k}+T_{s}\left(v_{set,k}+d_{v,k}\right)\sin\phi_{k},\\ \phi_{k+1} & =\phi_{k}+T_{s}\left(\omega_{set,k}+d_{\omega ,k}\right), \end{array}$$ (30)

where


$$\begin{array}{c} {𝐝}_{act,k}={\left[\begin{array}{cc} d_{v,k} & d_{\omega ,k} \end{array}\right]}^{T}. \end{array}$$ (31)

Equation (30) shows that the difference between the nominal MPC prediction model and the actual plant can be represented by a bounded actuator-side input mismatch when the command input, actuator saturation, and actuator dynamics are bounded. The holding-induced decision discrepancy in Eq (20) is treated separately because it arises from intermittent outer-loop optimization.

Accordingly, $y_{k}$ in Eq (27) and the predicted error terms used in the cost function are defined in the same reference-aligned local coordinates, thereby maintaining internal consistency of MPC prediction, cost evaluation, and feedback. Because wrap(·) is discontinuous at ±π, the linearization and optimization are intended to operate in a small-error region. When the heading error approaches the boundary of this region, an event-triggered update and re-linearization are used to restore the validity of the local prediction model.

### The trajectory tracking control problem: Formulation and analysis

This study addresses trajectory tracking of a differential-drive mobile robot under input-magnitude constraints, input-rate constraints, actuator saturation, and first-order actuator dynamics. Consistent with the previous subsection, the tracking error is evaluated in the reference-aligned local frame using $e_{k}$ defined in Eq (28).

At time *k*, the MPC predicts the future behavior over a finite prediction horizon of length *N*. The notation $(\cdot {)}_{k+i|k}$ denotes the *i*-step-ahead prediction made at time *k*, where i=0,1,…,N. In particular, $y_{k+i|k}$ denotes the predicted MPC output and $e_{k+i|k}$ denotes the predicted reference-aligned tracking error at step *k* + *i*, both computed based on information available at time *k*. In the prediction recursion, the propagated state corresponds to the LTV augmented world-frame error state $\xi_{k+i|k}$ generated by Eq (27), and the corresponding reference-aligned tracking error is obtained through the time-varying coordinate transformation along the reference trajectory:


$$\begin{array}{c} y_{k+i|k}=C_{k+i}\xi_{k+i|k},\;C_{k+i}=[\;T(\phi_{k+i}^{r})\;,\;0\;], \end{array}$$ (32)

where $\phi_{k+i}^{r}$ is the reference heading at step *k* + *i*. Because the transformation varies along the prediction horizon, the *i*-step prediction uses $T(\phi_{k+i}^{r})$ rather than a fixed $T(\phi_{k}^{r})$.

For compactness, the admissible set of the MPC velocity setpoint and the admissible set of the input increment are expressed as


$$\begin{array}{c} {𝐮}_{set,k}\in {𝒰}_{set},\;\Delta {𝐮}_{k}\in \Delta {𝒰}_{set}, \end{array}$$ (33)

where ${𝒰}_{set}$ and $\Delta {𝒰}_{set}$ denote the MPC setpoint and setpoint-increment constraint sets, respectively.

The trajectory-tracking control problem considered in this study can now be stated as follows. Given the reference trajectory ${𝐱}_{k}^{r}$ and the reference velocity ${𝐮}_{k}^{r}$, design a hierarchical controller that generates the outer-loop velocity setpoint ${𝐮}_{set,k}$, the actuator command ${𝐮}_{cmd,k}$, and the actual applied velocity ${𝐮}_{act,k}$, such that the following objectives are achieved:

1. The reference-aligned tracking error $e_{k}$ remains small and ultimately bounded.
2. The MPC velocity setpoint and input increment satisfy the constraints in Eq (33).
3. The actuator command satisfies the physical saturation limits.
4. The number of outer-loop MPC optimizations is reduced by updating the MPC only when the event-triggering condition is satisfied or when the maximum holding time is reached.
5. The holding-induced decision discrepancy and the actuator-side execution mismatch are explicitly distinguished. The former is regulated by the event-update policy, whereas the latter is reduced by the inner-loop velocity servo and evaluated in the stability and simulation analyses.

Periodic MPC typically achieves high tracking accuracy but requires solving an optimization problem at every sampling instant. By contrast, the event-triggered hierarchical MPC-PID framework developed in the next section introduces a tunable trade-off between computational efficiency and tracking performance. The modeling formulation above also clarifies that the proposed method is not designed for an ideal plant where the MPC input is applied instantaneously; instead, it addresses the more practical case where the robot is driven by delayed and saturated actuator velocities.

### Controller design

Building on the error-augmented discrete-time model developed in Section 2, this section presents the proposed event-triggered MPC–PID trajectory-tracking controller. The objective is to reduce unnecessary online MPC optimization while maintaining practically acceptable tracking performance in the presence of input constraints, actuator saturation, setpoint holding, and finite actuator response speed. The proposed contribution does not arise from the cascaded MPC–PID architecture alone. Instead, it lies in explicitly coupling an event-triggered constrained MPC outer loop with a fixed-period PID velocity servo and in characterizing the resulting execution mismatch caused by intermittent optimization, actuator lag, and saturation.

**Fig 2:**
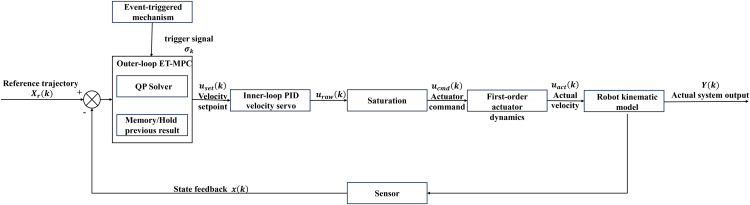
Overall architecture of the proposed ET–MPC–PID trajectory-tracking framework.

The overall architecture of the proposed ET–MPC–PID framework is shown in Fig 2. The outer-loop event-triggering mechanism determines whether the MPC optimization should be solved at the current sampling instant. When the triggering condition is not satisfied, the previously optimized velocity setpoint is retained by the memory unit. The inner-loop PID velocity servo operates at every sampling instant and regulates the actuator response toward the held MPC setpoint. Its raw output is first limited by the saturation block and subsequently filtered by the first-order actuator dynamics. Therefore, the velocity applied to the robot kinematic model is ${𝐮}_{act,k}$, rather than the nominal MPC setpoint ${𝐮}_{set,k}$.

All modules are implemented with the same sampling period $T_{s}$. The primary feedback signal is the reference-aligned geometric tracking error defined in Eq. (28). For the MPC outer loop, both the prediction output and the tracking-error term in the cost function are expressed in the reference-aligned local frame through the output mapping $C_{k}$ in Eq. (27). The velocity and input-rate constraints are consistent with the modeling definitions in Eqs. (9)–(11). The corresponding numerical parameters are listed in Table 3.

**Table 3:** Simulation, controller, and computational settings. Values in parentheses refer to the Eight scenario.

Parameter	Value
Sampling period	$T_{s}=0.02\;s$
State and input dimensions	$N_{x}=3$, $N_{u}=2$
Prediction and control horizons	$N_{p}=100$, $N_{c}=30$
MPC state-error weight	$Q_{a}=diag(1000,1000,1000)$; Eight: diag(900,900,1400)
MPC input-increment weight	$R_{a}=diag(120,140)$; Eight: diag(160,260)
Slack penalty	ρ=10
MPC setpoint bounds	$|v_{set}|\leq 3.5\;m/s$, $|\omega_{set}|\leq 3.5\;rad/s$
MPC input-rate bounds	$|\Delta v_{set}|\leq 3.0\;m/s$, $|\Delta \omega_{set}|\leq 3.0\;rad/s$ per sample
Actuator model	First-order velocity dynamics with $(\tau_{v},\tau_{\omega })=(0.20,0.15)\;s$
Physical command saturation bounds	$|v_{cmd}|\leq 15.0\;m/s$, $|\omega_{cmd}|\leq 2.0\;rad/s$
Inner-loop PID gains	$\left(K_{p,v},K_{i,v},K_{d,v})=(2,8,0\right)$, $\left(K_{p,\omega },K_{i,\omega },K_{d,\omega })=(2,6,0\right)$; conditional integration
Common nominal ET configuration	$J_{thr}=0.90$, $T_{\min}=0.08\;s$, $T_{\max}=0.12\;s$; curvature trigger disabled
Trigger normalization scales	$\varepsilon =[0.25\;m,5^{\circ },0.40\;m/s,4^{\circ }/s,6^{\circ }{]}^{T}$
Trigger-weight matrix	𝐖=diag(1.0,1.4,0.5,0.8,1.0)
VH–ET–MPC settings	η=0.80; $T_{\min}^{VH}=0.04\;s$, $T_{\max}^{VH}=0.12\;s$; position/heading guards $0.35\;m/7^{\circ }$; minimum/refresh horizons 60/64
Sensitivity ranges	$J_{thr}\in \{0.6,0.9,1.2,1.5,1.8\}$, $T_{\max}\in \{0.08,0.12,0.16,0.22,0.30\}\;s$
ET–MPC-retuned configuration	Double: $J_{thr}=0.86$, $T_{\min}=0.02\;s$, $T_{\max}=0.09\;s$; Eight: $J_{thr}=0.82$, $T_{\min}=0.02\;s$, $T_{\max}=0.08\;s$
QP solver	MATLAB quadprog, interior-point-convex algorithm; no warm start
QP dimensions	61 decision variables (60 control increments and one slack variable), 120 cumulative-input inequalities, and box constraints
Computing environment	MATLAB R2024b under Windows; Intel Core Ultra 5 225H CPU (1.70 GHz); 32 GB RAM

## ET–MPC–PID

### Outer-loop MPC optimization

The outer-loop MPC adopts the error-augmented LTV model in Eq. (27) as the prediction model. The prediction output ${𝐲}_{k}=C_{k}\xi_{k}$ corresponds to the reference-aligned tracking error, and the control increment $\Delta {𝐮}_{k}$ is selected as the optimization variable. This formulation enables explicit incorporation of input-rate constraints and promotes smooth velocity-setpoint generation.

Let the prediction horizon and the control horizon be $N_{p}$ and $N_{c}$, respectively, with $N_{c}\leq N_{p}$. The stacked prediction output and control-increment vectors are defined as


$$\begin{array}{c} 𝐘=[{𝐲}_{k+1|k}^{T},{𝐲}_{k+2|k}^{T},\ldots ,{𝐲}_{k+N_{p}|k}^{T}{]}^{T},\;\Delta 𝐔=[\Delta {𝐮}_{k|k}^{T},\Delta {𝐮}_{k+1|k}^{T},\ldots ,\Delta {𝐮}_{k+N_{c}-1|k}^{T}{]}^{T}. \end{array}$$ (34)

By recursively propagating the augmented model along the reference trajectory, the prediction relationship is written as


$$\begin{array}{c} 𝐘=\Phi_{k}\xi_{k}+\Theta_{k}\Delta 𝐔, \end{array}$$ (35)

where $\Phi_{k}$ and $\Theta_{k}$ are constructed from $A_{k}$, $B_{k}$, and $C_{k}$ along the prediction horizon.

The MPC performance index is selected as


$$\begin{array}{c} J_{k}=\sum_{i=1}^{N_{p}}{\left\|{𝐲}_{k+i|k}\right\|}_{𝐐}^{2}+\sum_{i=0}^{N_{c}-1}{\left\|\Delta {𝐮}_{k+i|k}\right\|}_{𝐑}^{2}+\rho \varepsilon^{2}, \end{array}$$ (36)

where 𝐐⪰0 and 𝐑≻0 are the weighting matrices for the tracking error and the input increment, respectively. The slack variable ε is introduced to improve numerical feasibility under constrained operating conditions, and ρ>0 is its penalty coefficient.

Combining Eq. (35) with the input and input-rate constraints gives the following quadratic program:


$$\begin{array}{cc} \min_{\Delta 𝐔,\;\varepsilon }\; & J_{k}\\ s.t.\; & {𝐮}_{set,k+i|k}\in [{𝐮}_{set,min},\;{𝐮}_{set,max}],\;i=0,\ldots ,N_{p}-1,\\ & \Delta {𝐮}_{k+i|k}\in [\Delta {𝐮}_{set,min},\;\Delta {𝐮}_{set,max}],\;i=0,\ldots ,N_{c}-1,\\ & \varepsilon \geq 0. \end{array}$$ (37)

The predicted velocity setpoint satisfies


$$\begin{array}{c} {𝐮}_{set,k+i|k}={𝐮}_{set,k+i-1|k}+\Delta {𝐮}_{k+i-1|k},\;i\geq 1. \end{array}$$ (38)

The constrained receding-horizon formulation in Eq. (37) follows standard stabilizing MPC principles, in which input constraints, input-rate constraints, feasibility, and local closed-loop properties are considered within a unified optimization framework [28,29]. In the present implementation, the same QP structure is used for the periodic MPC benchmark and for the event-triggered outer loop. Their difference lies only in the update schedule of the outer-loop optimization.

After solving the QP, only the first optimal control increment is implemented:


$$\begin{array}{c} {𝐮}_{set,k}=[\begin{array}{c} v_{set,k}\\ \omega_{set,k} \end{array}]={𝐮}_{set,k-1}+\Delta {𝐮}_{k|k}^{*}. \end{array}$$ (39)

For compact notation, the MPC solution mapping is denoted by


$$\begin{array}{c} ℳ\left(\xi_{k}\right)≜{𝐮}_{set,k}, \end{array}$$ (40)

where ℳ(·) denotes solving the QP in Eq. (37) and extracting the first velocity setpoint.

The MPC prediction model optimizes the nominal setpoint ${𝐮}_{set,k}$, whereas the physical robot is driven by ${𝐮}_{act,k}$. Their difference is retained explicitly in the following inner-loop design and practical boundedness analysis.

### Inner-loop PID execution and actuator model

The inner loop regulates the actuator response toward the outer-loop MPC setpoint. The velocity tracking error is defined as


$$\begin{array}{c} {𝐞}_{u,k}={𝐮}_{set,k}-{𝐮}_{act,k}. \end{array}$$ (41)

A discrete-time PID-form velocity servo generates the actuator command:


$$\begin{array}{c} {𝐮}_{raw,k}={𝐊}_{P}^{u}{𝐞}_{u,k}+{𝐊}_{I}^{u}{𝐈}_{u,k}+{𝐊}_{D}^{u}\frac{{𝐞}_{u,k}-{𝐞}_{u,k-1}}{T_{s}}, \end{array}$$ (42)

where ${𝐊}_{P}^{u}$, ${𝐊}_{I}^{u}$, and ${𝐊}_{D}^{u}$ are the proportional, integral, and derivative gain matrices, respectively, and ${𝐈}_{u,k}$ is the integral state.

The actuator command is obtained through component-wise physical saturation:


$$\begin{array}{c} {𝐮}_{cmd,k}={sat}_{{𝒰}_{cmd}}\left({𝐮}_{raw,k}\right),\;{𝐮}_{cmd,min}\leq {𝐮}_{cmd,k}\leq {𝐮}_{cmd,max}. \end{array}$$ (43)

To mitigate integrator windup, the implementation uses conditional integration rather than a back-calculation gain. For each velocity channel j∈{v,ω}, the integral state is updated as


$$\begin{array}{c} I_{j,k+1}=\left\{ \begin{array}{cc} I_{j,k}, & u_{raw,j,k}>u_{cmd,max,j}\;\wedge \;e_{j,k}>0,\\ I_{j,k}, & u_{raw,j,k}<u_{cmd,min,j}\;\wedge \;e_{j,k}<0,\\ I_{j,k}+T_{s}e_{j,k}, & otherwise. \end{array}\right. \end{array}$$ (44)

The integral state is therefore held whenever the unsaturated command would drive the actuator further into an active saturation limit; otherwise, conventional discrete integration is applied. The reported derivative gains are zero, so the implemented controller is a PI specialization of the PID-form expression in Eq. (42).

The actuator dynamics are modeled as


$$\begin{array}{c} {𝐮}_{act,k+1}=A_{u}{𝐮}_{act,k}+\left({𝐈}_{2}-A_{u}\right){𝐮}_{cmd,k}, \end{array}$$ (45)

where $A_{u}$ is defined in Eq. (18), and ${𝐈}_{2}$ denotes the 2×2 identity matrix. This model represents the finite response bandwidth of the linear- and angular-velocity channels. Consequently, even when the nominal MPC setpoint satisfies the optimization constraints, the actual actuator velocity can lag behind the setpoint because of actuator dynamics and command saturation.

For the no-PID event-triggered ablation controller, denoted as ET–MPC-same, the command input is directly obtained from the MPC setpoint:


$$\begin{array}{c} {𝐮}_{cmd,k}={sat}_{{𝒰}_{cmd}}\left({𝐮}_{set,k}\right). \end{array}$$ (46)

The same first-order actuator model in Eq. (45) is retained. Therefore, the comparison between ET–MPC-same and ET–MPC–PID isolates the contribution of the fixed-period PID velocity servo under identical nominal MPC settings, triggering parameters, actuator dynamics, and physical constraints.

### Event-triggering mechanism

The event-triggering mechanism determines the update instants of the outer-loop MPC. Instead of solving the QP at every sampling instant, the MPC is updated only when the accumulated mismatch relative to the latest optimization instant becomes sufficiently large or when a prescribed maximum holding duration is reached. During each inter-event interval, the outer-loop setpoint is held constant, while the PID–actuator channel continues to operate at the fixed sampling period.

Let $k_{i}$ denote the most recent outer-loop optimization instant. The triggering vector is defined as


$$\begin{array}{c} {𝐞}_{trig}(k)=[\Delta e_{pos}(k),\;\Delta e_{\phi }(k),\;\Delta v_{r}(k),\;\Delta \omega_{r}(k),\;\Delta \phi_{r}(k){]}^{T}. \end{array}$$ (47)

The first two components describe changes in tracking error relative to $k_{i}$:


$$\begin{array}{c} \Delta e_{pos}(k)=e_{pos}(k)-e_{pos}(k_{i}),\;\Delta e_{\phi }(k)=wrap\left(e_{\phi }(k)-e_{\phi }(k_{i})\right), \end{array}$$ (48)

where


$$\begin{array}{c} e_{pos}(k)=\sqrt{e_{x}^{2}(k)+e_{y}^{2}(k)}. \end{array}$$ (49)

The remaining components quantify reference variation:


$$\begin{array}{c} \Delta v_{r}(k)=v_{r}(k)-v_{r}(k_{i}),\;\Delta \omega_{r}(k)=\omega_{r}(k)-\omega_{r}(k_{i}), \end{array}$$ (50)

and


$$\begin{array}{c} \Delta \phi_{r}(k)=wrap\left(\phi_{r}(k)-\phi_{r}(k_{i})\right). \end{array}$$ (51)

Here, ${𝐞}_{k}=[e_{x}(k),e_{y}(k),e_{\phi }(k){]}^{T}$ is the reference-aligned tracking error defined in Eq. (28). By construction, ${𝐞}_{trig}(k_{i})=0$.

To prevent a single component from dominating the triggering decision solely because of its numerical scale, the normalization vector is defined as


$$\begin{array}{c} \varepsilon =[\varepsilon_{pos},\;\varepsilon_{\phi },\;\varepsilon_{v_{r}},\;\varepsilon_{\omega_{r}},\;\varepsilon_{\phi_{r}}{]}^{T}, \end{array}$$ (52)

with normalization matrix


$$\begin{array}{c} 𝐃=diag\left(\frac{1}{\varepsilon_{pos}},\frac{1}{\varepsilon_{\phi }},\frac{1}{\varepsilon_{v_{r}}},\frac{1}{\varepsilon_{\omega_{r}}},\frac{1}{\varepsilon_{\phi_{r}}}\right). \end{array}$$ (53)

A diagonal weighting matrix is introduced to adjust the relative importance of the normalized components:


$$\begin{array}{c} 𝐖=diag\left(w_{pos},w_{\phi },w_{\Delta v},w_{\Delta \omega },1\right). \end{array}$$ (54)

The scalar normalized triggering metric is


$$\begin{array}{c} g(k)={\left\|𝐖𝐃{𝐞}_{trig}(k)\right\|}_{\infty }. \end{array}$$ (55)

The basic triggering condition is written as


$$\begin{array}{c} g(k)\geq \delta , \end{array}$$ (56)

where δ>0, denoted as $J_{thr}$ in the numerical implementation, is the global normalized trigger threshold. A smaller δ generally causes more frequent MPC updates and lower holding-induced mismatch, whereas a larger δ reduces the optimization call rate at the cost of a potentially larger tracking neighborhood.

To avoid both excessively frequent updates and unbounded holding intervals, a minimum inter-event time $T_{\min}$ and maximum holding time $T_{\max}$ are imposed:


$$\begin{array}{c} N_{\min}=\left\lceil \frac{T_{\min}}{T_{s}}\right\rceil ,\;N_{\max}=\left\lceil \frac{T_{\max}}{T_{s}}\right\rceil . \end{array}$$ (57)

The complete outer-loop update law is


$$\begin{array}{c} {𝐮}_{set}(k)=\left\{ \begin{array}{cc} ℳ\left(\xi (k)\right), & [g(k)\geq \delta \;\wedge \;(k-k_{i})\geq N_{\min}]\;\vee \;(k-k_{i})\geq N_{\max},\\ {𝐮}_{set}(k_{i}), & otherwise. \end{array}\right. \end{array}$$ (58)

Whenever an update occurs, $k_{i}\leftarrow k$. The forced-update condition guarantees that the outer-loop MPC is re-solved within a bounded number of sampling steps even when the normalized triggering metric remains below the threshold.

The selection of $T_{\min}$ and $T_{\max}$ should be coordinated with the response capability of the inner-loop PID–actuator channel. Let $\tau_{cl}$ denote an equivalent closed-loop response time constant. The velocity tracking error can be approximated by


$$\begin{array}{c} \left\|{𝐞}_{u}(t)\right\|\leq \left\|{𝐞}_{u}(t_{i})\right\|\exp\left(-\frac{t-t_{i}}{\tau_{cl}}\right)+{\bar{d}}_{u}, \end{array}$$ (59)

where ${\bar{d}}_{u}$ represents the residual contribution of saturation, actuator lag, and unmodeled dynamics. A practical tuning guideline is


$$\begin{array}{c} T_{\min}\geq \tau_{cl}\ln\left(\frac{1}{\gamma }\right),\;T_{\max}\leq \frac{{\bar{e}}_{hold}}{L_{r}}, \end{array}$$ (60)

where γ∈(0,1) is the desired decay ratio, $L_{r}$ is an upper bound on the reference-variation rate, and ${\bar{e}}_{hold}$ denotes the allowable holding-induced error.

For reproducibility, $\tau_{cl}$ is operationally defined as the larger 95% settling time of the linear- and angular-velocity channels in the closed-loop PID–actuator step response. The reference-variation bound is calculated from the sampled reference trajectory as


$$\begin{array}{c} L_{r}=\max_{k}{\left\|[\begin{array}{c} \frac{v_{k+1}^{r}-v_{k}^{r}}{T_{s}}\\ \frac{\omega_{k+1}^{r}-\omega_{k}^{r}}{T_{s}}\\ \frac{\mathrm{wrap}\left(\phi_{k+1}^{r}-\phi_{k}^{r}\right)}{T_{s}} \end{array}]\right\|}_{2}. \end{array}$$ (61)

The quantity ${\bar{e}}_{hold}$ represents a prescribed additional tracking-error budget attributable to retaining an outdated MPC decision. It is used as an engineering tolerance for interpreting the maximum permissible holding interval rather than as an independently optimized controller parameter.

These relations are practical coordination guidelines rather than necessary stability conditions or an unreported analytical parameter-fitting rule. In the reported simulations, they were used as preliminary consistency checks for the inner-loop response and reference variation. The final nominal trigger configuration was selected from the two-parameter sensitivity and Pareto study in Section 4, which explicitly reports the accuracy–computation trade-off.

For the final controller comparison, the same nominal triggering configuration is used for ET–MPC-same and ET–MPC–PID in both trajectories. No auxiliary curvature-trigger term is activated in the sensitivity study or in the final nominal comparison; therefore, the reported update-frequency trade-off is determined directly by $J_{thr}$ and $T_{\max}$.

### External VH–ET–MPC reference implementation

To distinguish the contribution of the proposed fixed-period PID velocity servo from the influence of the event-update policy itself, an external variable-horizon event-triggered MPC reference, denoted as VH–ET–MPC, is implemented following the event-triggered predictive-control principles reported in [24]. This controller is not an ablation of the proposed architecture. Instead, it serves as an external reference method with a distinct event-triggering and stored-sequence execution policy.

At each VH–ET–MPC optimization instant, a constrained MPC problem is solved and the resulting optimal input sequence is stored. Between two optimization events, the stored sequence is advanced and its next element is applied rather than recomputing a new control sequence at every sampling instant. The event condition is based on a normalized state-prediction mismatch between the current closed-loop state and the state predicted from the most recent optimization. The prediction horizon contracts as the stored sequence is executed and is refreshed before the remaining horizon becomes excessively short.

For a same-platform comparison, the VH–ET–MPC reference uses the differential-drive model, sampling period, reference trajectories, QP solver, input bounds, input-rate bounds, and actuator model adopted in this study. It does not include the fixed-period PID velocity servo of the proposed ET–MPC–PID controller. A single normalized mismatch threshold is used for both trajectories. In addition, a minimum dwell time, a bounded open-loop interval, and position and heading error guards are imposed to maintain the comparison within a feasible operating region. The variable-horizon parameters and safeguard values are listed in Table 3.

The VH–ET–MPC reference therefore retains the characteristic prediction-mismatch triggering, stored-input-sequence execution, and contracting-horizon mechanism of the external method, while the plant-side model and physical constraints remain identical to those used for the other MPC-type controllers. This implementation prevents differences in robot model, actuator dynamics, numerical solver, or constraint set from being misinterpreted as differences caused by the event-triggered control policy.

### Stability analysis

This subsection provides a local practical boundedness characterization for the proposed ET–MPC–PID controller and its internal no-PID ablation variants. It is not intended as a stability proof for the external VH–ET–MPC reference controller, whose event rule and stored-sequence execution mechanism differ from the proposed structure. Because the proposed event-triggering mechanism holds the MPC setpoint over inter-event intervals and the actual robot input generally differs from the nominal MPC setpoint, strict asymptotic convergence to the origin is not expected in general. The closed-loop tracking error is therefore analyzed in the sense of local ultimate boundedness. The analysis follows local MPC arguments, piecewise-affine representations of constrained MPC, and Lyapunov/BIBO boundedness tools [28–32].

**Assumption 1 (Operating region and local prediction validity).** The closed-loop trajectory remains in a neighborhood of the reference trajectory in which the error-augmented LTV prediction model in Eq. (27) is valid. Let ${𝐫}_{lin,k}$ denote the residual of the local linear approximation. It is assumed that


$$\begin{array}{c} \left\|{𝐫}_{lin,k}\right\|\leq {\bar{w}}_{lin} \end{array}$$ (62)

for a finite constant ${\bar{w}}_{lin}>0$ throughout this local operating region.

**Assumption 2 (Local equivalent feedback form of nominal periodic MPC).** Within the operating region, the periodic MPC controller with the QP formulation in Eq. (37) is locally stabilizing. When the active constraint set remains locally constant or switches finitely many times, the constrained MPC law admits a local piecewise-affine state-feedback representation [30,31]. Therefore, the nominal closed-loop error dynamics can be expressed as


$$\begin{array}{c} \xi_{k+1}={𝐀}_{cl}\xi_{k},\;\rho \left({𝐀}_{cl}\right)<1, \end{array}$$ (63)

where ${𝐀}_{cl}$ is Schur stable.

**Assumption 3 (Local recursive feasibility).** The outer-loop MPC problem is feasible initially and remains feasible within the operating region under standard constrained MPC conditions [28,29]. This is a local assumption consistent with the finite-horizon QP in Eq. (37).

**Assumption 4 (Bounded PID–actuator execution mismatch).** For the selected PID gains, bounded setpoints, bounded input increments, actuator saturation, and stable first-order actuator dynamics, the local PID–actuator channel has bounded input–bounded output behavior. Consequently, the execution mismatch


$$\begin{array}{c} {𝐝}_{act,k}={𝐮}_{act,k}-{𝐮}_{set,k} \end{array}$$ (64)

is bounded:


$$\begin{array}{c} \left\|{𝐝}_{act,k}\right\|\leq {\bar{d}}_{act}. \end{array}$$ (65)

This condition is consistent with standard BIBO arguments for stable systems with bounded inputs and saturation [32].

**Assumption 5 (Bounded holding-induced perturbation).** Within the operating region, the reference trajectory and the local MPC solution map are Lipschitz continuous. Under the triggering condition in Eq. (56) and the bounded holding interval in Eq. (57), there exist nonnegative constants $c_{\delta }$ and $c_{T}$ such that the perturbation induced by holding the previous MPC setpoint satisfies


$$\begin{array}{c} \left\|{𝐰}_{hold,k}\right\|\leq c_{\delta }\delta +c_{T}T_{\max}. \end{array}$$ (66)

Under the update law in Eq. (58), any two consecutive optimization instants $k_{i}$ and $k_{i+1}$ satisfy


$$\begin{array}{c} 1\leq k_{i+1}-k_{i}\leq N_{\max}. \end{array}$$ (67)

The upper bound follows directly from the forced-update condition. Hence, the outer-loop holding duration is bounded, which prevents indefinitely long open-loop execution of an outdated MPC setpoint.

The equivalent perturbation affecting the nominal closed-loop error dynamics is decomposed as


$$\begin{array}{c} {𝐰}_{k}={𝐰}_{hold,k}+{𝐰}_{lag,k}+{𝐰}_{sat,k}+{𝐫}_{lin,k}, \end{array}$$ (68)

where ${𝐰}_{hold,k}$ is induced by retaining an outdated outer-loop decision, ${𝐰}_{lag,k}$ is caused by actuator lag, ${𝐰}_{sat,k}$ is caused by command saturation, and ${𝐫}_{lin,k}$ is the local linearization residual. Under the preceding assumptions, there exists a finite constant $\bar{w}>0$ such that


$$\begin{array}{c} \left\|{𝐰}_{k}\right\|\leq \bar{w}\leq c_{\delta }\delta +c_{T}T_{\max}+{\bar{w}}_{lag}+{\bar{w}}_{sat}+{\bar{w}}_{lin}. \end{array}$$ (69)

The perturbed local closed-loop dynamics can therefore be expressed as


$$\begin{array}{c} \xi_{k+1}={𝐀}_{cl}\xi_{k}+{𝐁}_{w}{𝐰}_{k}, \end{array}$$ (70)

where ${𝐁}_{w}$ is the disturbance input matrix. Equation (70) represents the proposed controller as a nominal locally stabilizing MPC loop perturbed by bounded setpoint-holding, actuator-lag, saturation, and local linearization-residual effects.

Since ${𝐀}_{cl}$ is Schur stable, for any ${𝐐}_{s}≻0$, there exists a unique ${𝐏}_{s}≻0$ satisfying [32]


$$\begin{array}{c} {𝐀}_{cl}^{T}{𝐏}_{s}{𝐀}_{cl}-{𝐏}_{s}=-{𝐐}_{s}. \end{array}$$ (71)

Choose the Lyapunov candidate


$$\begin{array}{c} V_{k}=\xi_{k}^{T}{𝐏}_{s}\xi_{k}. \end{array}$$ (72)

Combining Eqs. (70) and (71) gives


$$\begin{array}{cc} V_{k+1}-V_{k}= & -\xi_{k}^{T}{𝐐}_{s}\xi_{k}+2\xi_{k}^{T}{𝐀}_{cl}^{T}{𝐏}_{s}{𝐁}_{w}{𝐰}_{k}\\ & +{𝐰}_{k}^{T}{𝐁}_{w}^{T}{𝐏}_{s}{𝐁}_{w}{𝐰}_{k}. \end{array}$$ (73)

Define


$$\begin{array}{c} c_{1}=\lambda_{\min}\left({𝐐}_{s}\right),\;c_{2}=\left\|{𝐀}_{cl}^{T}{𝐏}_{s}{𝐁}_{w}\right\|,\;c_{3}=\left\|{𝐁}_{w}^{T}{𝐏}_{s}{𝐁}_{w}\right\|. \end{array}$$ (74)

By Young’s inequality, for any η>0,


$$\begin{array}{c} 2\xi_{k}^{T}{𝐀}_{cl}^{T}{𝐏}_{s}{𝐁}_{w}{𝐰}_{k}\leq \eta {\left\|\xi_{k}\right\|}^{2}+\frac{c_{2}^{2}}{\eta }{\left\|{𝐰}_{k}\right\|}^{2}. \end{array}$$ (75)

Substituting Eq. (75) into Eq. (73) yields


$$\begin{array}{c} V_{k+1}-V_{k}\leq -(c_{1}-\eta ){\left\|\xi_{k}\right\|}^{2}+\left(\frac{c_{2}^{2}}{\eta }+c_{3}\right){\left\|{𝐰}_{k}\right\|}^{2}. \end{array}$$ (76)

Choose $0<\eta <c_{1}$ and define


$$\begin{array}{c} a=c_{1}-\eta >0,\;b=\frac{c_{2}^{2}}{\eta }+c_{3}>0. \end{array}$$ (77)

Then


$$\begin{array}{c} V_{k+1}-V_{k}\leq -a{\left\|\xi_{k}\right\|}^{2}+b{\left\|{𝐰}_{k}\right\|}^{2}. \end{array}$$ (78)

Using Eq. (69) gives


$$\begin{array}{c} V_{k+1}-V_{k}\leq -a{\left\|\xi_{k}\right\|}^{2}+b{\bar{w}}^{2}. \end{array}$$ (79)

Since ${𝐏}_{s}≻0$,


$$\begin{array}{c} \lambda_{\min}\left({𝐏}_{s}\right){\left\|\xi_{k}\right\|}^{2}\leq V_{k}\leq \lambda_{\max}\left({𝐏}_{s}\right){\left\|\xi_{k}\right\|}^{2}. \end{array}$$ (80)

Therefore, the Lyapunov function decreases whenever


$$\begin{array}{c} {\left\|\xi_{k}\right\|}^{2}>\frac{b}{a}{\bar{w}}^{2}. \end{array}$$ (81)

The resulting local practical boundedness condition is


$$\begin{array}{c} \underset{k\to \infty }{lim sup}\left\|\xi_{k}\right\|\leq \sqrt{\frac{b}{a}\cdot \frac{\lambda_{\max}\left({𝐏}_{s}\right)}{\lambda_{\min}\left({𝐏}_{s}\right)}}\bar{w}. \end{array}$$ (82)

Equation (82) establishes that the closed-loop error converges to a bounded neighborhood whose size depends on the aggregate perturbation $\bar{w}$. In particular, Eq. (69) clarifies the design trade-off: reducing $J_{thr}$ or $T_{\max}$ decreases the holding-induced component but increases the MPC update frequency; improving the PID–actuator response and avoiding persistent saturation reduce the actuator-related components; and maintaining operation within the local prediction region reduces the contribution of the linearization residual.

For the simulation ablation study, ET–MPC-same uses exactly the same nominal trigger threshold, minimum dwell time, maximum holding time, MPC formulation, constraints, actuator dynamics, and reference trajectory as ET–MPC–PID, while removing only the fixed-period PID velocity servo. ET–MPC-retuned also removes the PID servo but uses more aggressive event-trigger settings to increase the MPC update frequency. These internal comparisons examine whether the benefit of the proposed architecture can be reproduced by increasing MPC update frequency alone, or whether fixed-period velocity compensation remains necessary when the outer-loop optimization is intermittent.

The local practical boundedness result characterizes the deterministic bounded-perturbation model used in the controller analysis. The Monte Carlo study in Section 4 separately evaluates empirical robustness under paired sampled execution uncertainties, including initial-state deviations, actuator-parameter mismatch, measurement noise, and additive equivalent velocity disturbances. This empirical study complements the deterministic local characterization but does not constitute a probabilistic stability guarantee.

## Results and discussion

To evaluate the proposed ET–MPC–PID framework, comparative simulations were conducted for a differential-drive mobile robot under two representative reference trajectories. The Double scenario contains a double lane-change maneuver followed by a quasi-steady segment, whereas the Eight scenario contains repeated high-curvature motion and directional switching. The proposed controller was compared with six alternatives: conventional PID, periodic MPC, periodic MPC–PID, a variable-horizon event-triggered MPC benchmark (VH–ET–MPC), ET–MPC-same, and ET–MPC-retuned. Periodic MPC–PID provides a high-update hierarchical reference. VH–ET–MPC provides an external event-triggered predictive-control reference. ET–MPC-same uses exactly the same nominal event configuration as ET–MPC–PID but removes the fixed-period PID velocity servo, thereby isolating the effect of inner-loop actuator compensation. ET–MPC-retuned also removes the PID servo and deliberately uses a more aggressive update policy. It is included as a diagnostic ablation to determine whether increasing the MPC update frequency alone can compensate for the absence of inner-loop velocity regulation.

Within each scenario, all MPC-type controllers use the same robot model, reference trajectory, sampling period, prediction horizon, control horizon, state and input weights, input bounds, input-rate bounds, actuator limits, and quadratic-programming solver. Therefore, the comparison isolates the effect of the event-update policy and the fixed-period PID velocity servo. The nominal ET–MPC-same and ET–MPC–PID configurations use the common normalized trigger threshold $J_{thr}=0.90$, minimum holding time $T_{\min}=0.08\;s$, and maximum holding time $T_{\max}=0.12\;s$ for both trajectories. Curvature-triggering is disabled in this comparison so that the reported behavior is determined by the normalized trigger score and holding-time bound. The principal simulation, controller, and implementation settings are listed in Table 3.

The holding-time guideline in Section 3 was used as a preliminary consistency check rather than as a hidden parameter-fitting procedure. Specifically, $\tau_{cl}$ is defined as the larger 95% settling time of the two closed-loop PID–actuator velocity channels, and $L_{r}$ is calculated from the maximum sampled reference-variation rate in Eq. (61). The quantity ${\bar{e}}_{hold}$ represents a prescribed additional tracking-error budget associated with retaining an outdated outer-loop decision. The final nominal setting was selected through the two-parameter sensitivity and Pareto study, which directly reports the trade-off associated with $J_{thr}$ and $T_{\max}$. In the nominal ablation comparison, ET–MPC-same and ET–MPC–PID use the same common nominal trigger configuration in both scenarios. The retuned ET–MPC case is intentionally excluded from this equality because it is designed only to expose the computational consequence of increasing the update density.

**Fig 3:**
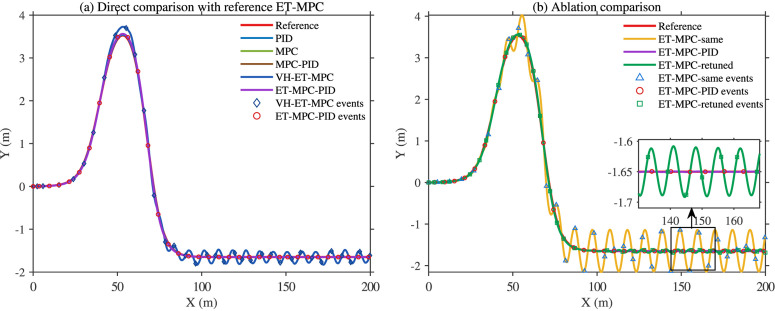
Trajectory tracking and ablation comparison under the Double scenario. (a) Direct comparison including the VH–ET–MPC benchmark. (b) Event-triggered ablation comparison. Markers indicate selected MPC update instants.

Fig 3 presents the tracking and ablation results in the Double scenario. In the direct comparison, periodic MPC, periodic MPC–PID, VH–ET–MPC, and ET–MPC–PID all follow the reference path without visible divergence. In the ablation comparison, ET–MPC-same exhibits oscillatory behavior after the lane-change maneuver. Since ET–MPC-same and ET–MPC–PID use identical event parameters, this difference is attributable to the presence of the fixed-period PID velocity servo rather than to a higher outer-loop update rate. ET–MPC-retuned reduces the visible oscillation through substantially more frequent optimization, but this reduction is accompanied by a much larger computational burden.

**Fig 4:**
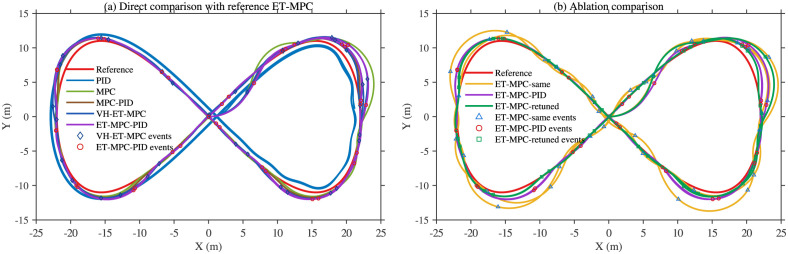
Trajectory tracking and ablation comparison under the Eight scenario. (a) Direct comparison including the VH–ET–MPC benchmark. (b) Event-triggered ablation comparison. Markers indicate selected MPC update instants.

The corresponding Eight-scenario results are shown in Fig 4. This trajectory imposes a more demanding tracking task because the robot repeatedly traverses high-curvature regions and changes direction. The PID controller exhibits visible deviations because it does not optimize future constrained motion. ET–MPC-same produces larger deviations in both loops of the figure-eight trajectory, indicating that directly holding the outer-loop MPC setpoint is insufficient when actuator lag and saturation effects are present. ET–MPC–PID substantially reduces these deviations while retaining a low event-update rate. ET–MPC-retuned improves the ET–MPC trajectory only by increasing the update density toward that of periodic MPC.

**Fig 5:**
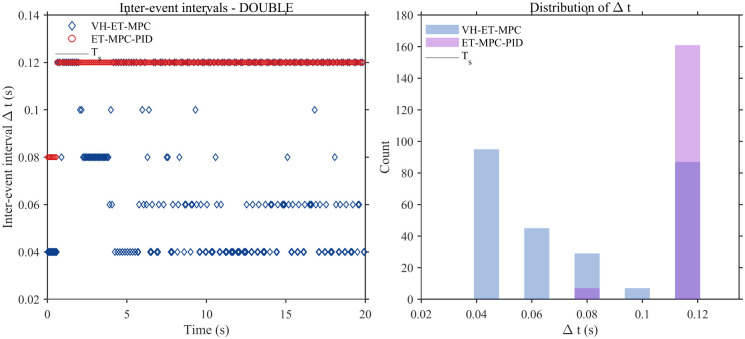
Inter-event intervals and distributions under the Double scenario. The results compare VH–ET–MPC and ET–MPC–PID.

**Fig 6:**
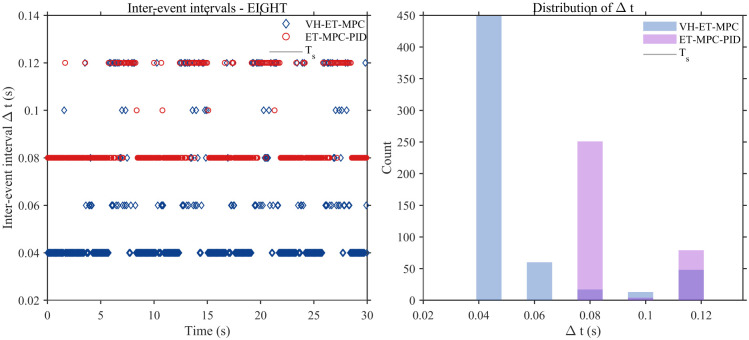
Inter-event intervals and distributions under the Eight scenario. The results compare VH–ET–MPC and ET–MPC–PID.

The inter-event behavior is examined in Figs 5 and 6. In the Double scenario, the ET–MPC–PID intervals are primarily concentrated at the maximum holding time of 0.12 s, indicating that the held setpoint remains adequate during extended low-demand portions of the trajectory. Shorter intervals occur when the normalized trigger score exceeds its threshold. In contrast, the VH–ET–MPC benchmark shows a broader distribution because its variable-horizon event policy follows a different update mechanism.

In the Eight scenario, the ET–MPC–PID intervals are more frequently concentrated near $T_{\min}=0.08\;s$, reflecting the larger tracking demand around repeated turns and directional transitions. Intervals of 0.10 s and 0.12 s still appear during less demanding portions of the path. These results show that the event mechanism adapts the optimization frequency to the local motion condition while maintaining a strictly positive lower bound on the inter-event interval.

**Fig 7:**
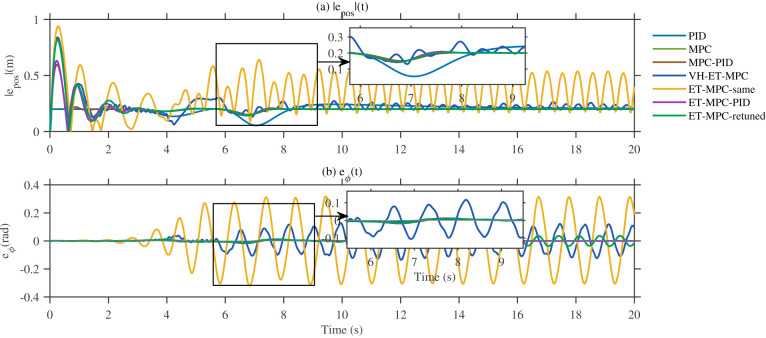
Tracking-error response under the Double scenario. Insets show enlarged local responses.

The time-domain errors in the Double scenario are shown in Fig 7. ET–MPC-same produces sustained position and heading oscillations after the main lane-change maneuver. The corresponding ET–MPC–PID response remains close to the periodic MPC-based responses under the same event-triggering configuration. This result indicates that sparse outer-loop optimization does not by itself lead to acceptable execution when the actuator chain is not compensated at the fixed control period.

**Fig 8:**
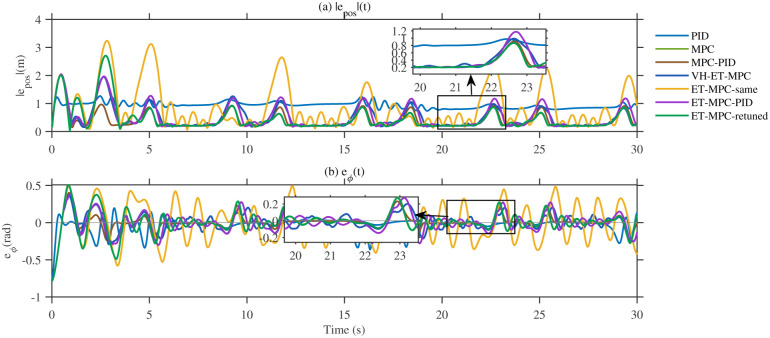
Tracking-error response under the Eight scenario. Insets show enlarged local responses.

Fig 8 shows the error responses in the Eight scenario. The ET–MPC-same controller produces the largest error peaks during curvature-intensive phases. ET–MPC–PID markedly reduces these peaks and maintains a smoother response despite using only 22.3% of the periodic MPC update rate in the nominal simulation. ET–MPC-retuned approaches the tracking behavior of the proposed controller, but it does so with an update rate of 98.3%, which largely removes the computational advantage associated with event-triggered optimization.

**Fig 9:**
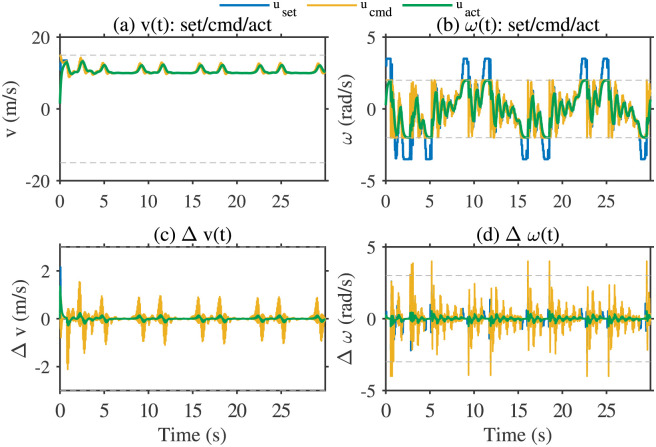
Closed-loop consistency and constraint verification for ET–MPC–PID under the Eight scenario. ${𝐮}_{set}$, ${𝐮}_{cmd}$, and ${𝐮}_{act}$ denote the MPC setpoint, saturated actuator command, and actual actuator velocity, respectively.

Fig 9 further examines the hierarchical execution chain for ET–MPC–PID in the Eight scenario. The optimized MPC setpoint ${𝐮}_{set}$, saturated actuator command ${𝐮}_{cmd}$, and actual actuator velocity ${𝐮}_{act}$ are distinct, confirming that the physical execution signal is not identical to the nominal outer-loop setpoint. The actual velocity follows the command with the expected first-order lag, while the command and actual actuator signals remain within the prescribed magnitude bounds. The input increments also remain bounded. This observation supports the actuator-mismatch formulation used in the model and local practical-boundedness characterization.

Table 4 summarizes the nominal tracking performance of all controllers. In the Double scenario, ET–MPC–PID achieves a position RMSE of 0.213 m, which is close to periodic MPC–PID (0.207 m) and substantially lower than ET–MPC-same (0.386 m). In the Eight scenario, ET–MPC–PID reduces the position RMSE from 1.158 m for ET–MPC-same to 0.661 m, while retaining a low QP call rate. The periodic MPC–PID controller provides the smallest nominal RMSE in the Eight scenario, but requires optimization at every sample. All QP-based controllers have zero solver failures and zero constraint-violation time steps under nominal conditions. The conventional PID controller produces 210 actuator-constraint violation time steps in the Eight scenario.

**Table 4:** Nominal tracking performance and online optimization demand.

Scenario	Method	Pos. RMSE (m)	Max pos. error (m)	Heading RMSE (rad)	Control variation	QP call rate (%)
Double	PID	0.203	0.243	0.003	0.002	–
Double	MPC	0.224	0.818	0.004	0.020	100.0
Double	MPC–PID	0.207	0.597	0.004	0.016	100.0
Double	VH–ET–MPC	0.243	0.840	0.065	0.138	26.4
Double	ET–MPC-same	0.386	0.939	0.186	0.149	24.0
Double	ET–MPC–PID	0.213	0.630	0.004	0.021	16.9
Double	ET–MPC-retuned	0.225	0.818	0.017	0.039	68.1
Eight	PID	0.944	1.269	0.104	0.121	–
Eight	MPC	0.625	2.708	0.144	0.096	100.0
Eight	MPC–PID	0.493	2.046	0.107	0.063	100.0
Eight	VH–ET–MPC	0.638	2.009	0.127	0.120	39.2
Eight	ET–MPC-same	1.158	3.238	0.254	0.155	24.9
Eight	ET–MPC–PID	0.661	2.038	0.139	0.107	22.3
Eight	ET–MPC-retuned	0.624	2.708	0.144	0.097	98.3

Control variation is defined as the sum of the mean absolute increments of linear and angular velocities. All QP-based controllers have zero QP failures and zero constraint-violation time steps under nominal conditions. The PID controller has 210 actuator-constraint violation time steps in the Eight scenario.

The computational profile is reported in Table 5. The QP runtime refers only to the optimization stage, whereas the trigger and PID timings refer to the measured routine execution time per sample. The trigger evaluation and fixed-period PID computations require less than 0.14 ms on average in all listed event-triggered cases. The mean QP time remains below the 20 ms sampling interval for all compared MPC-type controllers. However, the 95th-percentile and maximum QP times can exceed one sampling interval, particularly in the Eight scenario. Thus, the simulation confirms average computational compatibility with the chosen sampling period, but it does not establish a hard real-time guarantee for an embedded implementation. A compiled solver, warm start, and hardware-in-the-loop evaluation would be appropriate for a strict real-time deployment assessment.

**Table 5:** QP runtime, event-processing overhead, and solver status under nominal conditions.

Scenario	Method	QP calls	Call rate (%)	QP time: mean / P95 / max (ms)	Total QP time (s)	Trigger / PID time (ms)	QP failures
Double	MPC	1000	100.0	11.24 / 14.00 / 83.28	11.24	–	0
Double	MPC–PID	1000	100.0	11.92 / 15.08 / 33.39	11.92	– / 0.117	0
Double	VH–ET–MPC	264	26.4	9.74 / 15.12 / 20.21	2.57	0.042 / –	0
Double	ET–MPC-same	240	24.0	17.15 / 24.36 / 68.83	4.12	0.044 / –	0
Double	ET–MPC–PID	169	16.9	14.72 / 20.07 / 28.84	2.49	0.040 / 0.039	0
Double	ET–MPC-retuned	681	68.1	14.12 / 21.03 / 50.83	9.61	0.114 / –	0
Eight	MPC	1500	100.0	14.15 / 22.70 / 38.93	21.23	–	0
Eight	MPC–PID	1500	100.0	13.55 / 21.74 / 47.34	20.32	– / 0.114	0
Eight	VH–ET–MPC	588	39.2	12.11 / 20.81 / 39.24	7.12	0.051 / –	0
Eight	ET–MPC-same	374	24.9	19.02 / 26.94 / 34.40	7.11	0.043 / –	0
Eight	ET–MPC–PID	335	22.3	15.23 / 25.24 / 33.63	5.10	0.036 / 0.042	0
Eight	ET–MPC-retuned	1475	98.3	12.98 / 20.08 / 47.66	19.14	0.133 / –	0

Trigger and PID times are mean execution times per sample. The QP time is measured only for optimization calls. No warm-start strategy was used.

**Fig 10:**
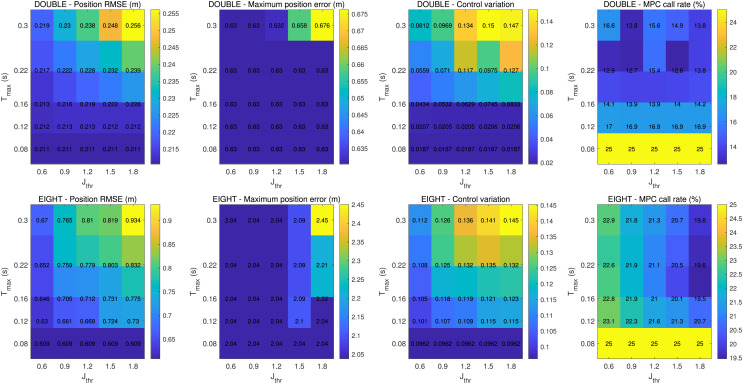
Sensitivity of ET–MPC–PID to the normalized trigger threshold and maximum holding time. The maps report position RMSE, maximum position error, control variation, and MPC call rate for the Double and Eight scenarios.

The sensitivity results in Fig 10 quantify the influence of the normalized trigger threshold $J_{thr}$ and the maximum holding time $T_{\max}$. The displayed grid contains 25 common parameter combinations for each trajectory. Smaller $J_{thr}$ or smaller $T_{\max}$ leads to more frequent MPC updates and generally lower tracking error. Increasing either parameter reduces the call rate but can increase position RMSE, maximum position error, and control variation. This trend is particularly evident for the Eight trajectory, where large holding times lead to a more pronounced loss of tracking accuracy. All grid points remain free of QP failures and constraint violations in the nominal simulations.

**Fig 11:**
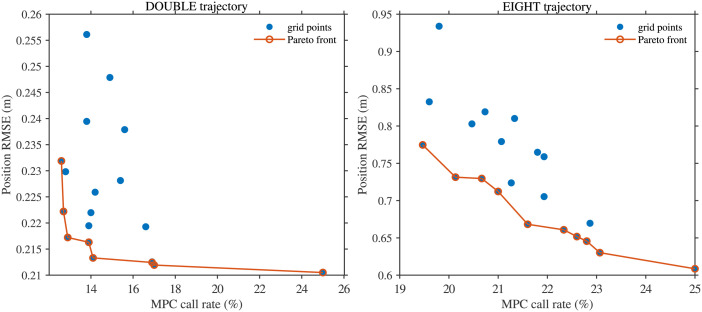
Pareto trade-off between position RMSE and MPC call rate. Each point corresponds to one $\left(J_{thr},T_{\max}\right)$ combination in the sensitivity grid.

Fig 11 presents the resulting accuracy–computation trade-off. The common nominal configuration $\left(J_{thr},T_{\max})=(0.90,0.12\;s\right)$ was selected as a shared compromise rather than as an independently optimized setting for each trajectory. In the Double scenario, it reduces the MPC call rate from 25.0% at (0.6,0.08 s) to 16.9%, while the position RMSE changes only from 0.211 m to 0.213 m. In the Eight scenario, the same configuration reduces the call rate from 25.0% to 22.3%, with the position RMSE changing from 0.609 m to 0.661 m. These results support the use of a common parameter setting while making the resulting accuracy–computation compromise explicit.

A paired Monte Carlo study was further conducted to evaluate empirical robustness under sampled execution uncertainty. Each scenario contains 100 trials, and every compared controller within a trial uses the same initial-state perturbation, actuator realization, measurement-noise sequence, and disturbance realization. The initial position and heading offsets are sampled from zero-mean Gaussian distributions with standard deviations of 0.06 m, 0.06 m, and $3^{\circ }$, respectively. The actuator time constants and gains are randomly perturbed and clipped to [0.65,1.35] and [0.82,1.18], respectively. Position and heading measurements are corrupted by zero-mean Gaussian noise with standard deviations of 0.015 m and $0.75^{\circ }$, respectively. Additive equivalent linear- and angular-velocity disturbance sequences with standard deviations of 0.030 m/s and 0.035 rad/s are injected into the actuator chain. A trial is defined as feasible when all logged signals are finite, no QP failure occurs, and no constraint-violation time step is recorded. Terminal success additionally requires a final position error not exceeding 1.0 m. This Monte Carlo study is an empirical robustness assessment and is not a probabilistic extension of the deterministic local boundedness result.

The Monte Carlo results are summarized in Table 6. In the Double scenario, ET–MPC–PID achieves 100% feasibility and terminal success, with a mean position RMSE of 0.220 m, a worst maximum position error of 1.048 m, and a mean MPC call rate of 18.0%. In the Eight scenario, ET–MPC–PID achieves 99% feasibility and 91% terminal success, compared with 33% terminal success for ET–MPC-same. Its worst maximum position error is 6.260 m, whereas ET–MPC-same reaches 21.153 m under the paired uncertainty trials. The proposed controller records one QP failure over the 100 Eight-scenario trials and no constraint-violation time step. This result indicates that the fixed-period PID velocity servo substantially improves robustness relative to ET–MPC-same under the same family of event-triggered execution conditions, while retaining a markedly lower optimization demand than periodic MPC–PID.

**Table 6:** Monte Carlo robustness results under paired execution uncertainty (100 trials per scenario).

Scenario	Method	Feasible / success (%)	Pos. RMSE (mean ± std; P95) (m)	Worst max. pos. error (m)	MPC call rate (%)	QP failures / violation steps
Double	MPC–PID	100 / 100	0.212±0.013; 0.236	1.047	100.00	0 / 0
Double	VH–ET–MPC	100 / 100	0.260±0.060; 0.346	1.481	26.64	0 / 0
Double	ET–MPC-same	100 / 100	0.412±0.083; 0.557	1.587	24.85	0 / 0
Double	ET–MPC–PID	100 / 100	0.220±0.013; 0.245	1.048	18.01	0 / 0
Eight	MPC–PID	100 / 97	0.573±0.153; 0.830	4.192	100.00	0 / 0
Eight	VH–ET–MPC	100 / 85	0.750±0.241; 1.138	4.759	39.75	0 / 0
Eight	ET–MPC-same	100 / 33	2.448±1.687; 5.431	21.153	24.93	0 / 0
Eight	ET–MPC–PID	99 / 91	0.742±0.274; 1.166	6.260	22.62	1 / 0

Feasible: finite logged signals, zero QP failures, and zero constraint-violation time steps. Success: feasible trial with final position error not exceeding 1.0 m. Mean ± standard deviation and P95 values are calculated from finite metric records. Worst max. position error is the largest sample-wise position error observed across all finite trial records for the corresponding method and scenario. Feasibility and success rates are evaluated over all 100 trials.

Overall, the results support three conclusions. First, event-triggered MPC without fixed-period inner-loop velocity compensation can accumulate holding-induced and actuator-lag-related mismatch, producing oscillatory or inaccurate tracking under sparse updates. Second, the proposed ET–MPC–PID controller mitigates this mismatch and preserves substantially better tracking behavior than ET–MPC-same under the same nominal event configuration. Third, increasing the event-update frequency can improve the behavior of ET–MPC without PID compensation, but the resulting QP demand can approach that of periodic MPC. These observations are consistent with the local practical-boundedness characterization in Section 3: reducing the holding-induced perturbation through tighter triggering improves tracking at the cost of more frequent optimization, whereas the fixed-period PID velocity servo reduces actuator-related mismatch without requiring the same increase in outer-loop QP calls. The present evidence is limited to simulation-based evaluation and practical boundedness under the stated assumptions; hardware validation and strict hard-real-time implementation remain important directions for future work.

## Conclusions and perspectives

This study developed an ET–MPC–PID trajectory-tracking framework for differential-drive mobile robots. The method combines an event-triggered constrained MPC outer loop with a fixed-period PID-form velocity servo, while explicitly distinguishing holding-induced decision discrepancy from actuator-side execution mismatch.

The local analysis characterizes practical boundedness under bounded holding-related deviation, actuator-side mismatch, and linearization residual. Simulations show that ET–MPC–PID achieves substantially better tracking than ET–MPC-same under the same nominal triggering configuration. Increasing the MPC update frequency can partly improve the no-PID event-triggered controller, but the associated QP demand approaches that of periodic MPC. Sensitivity, Pareto, runtime, and paired Monte Carlo analyses further demonstrate the accuracy–computation trade-off and empirical robustness under sampled execution uncertainty.

Future work will consider physical robot experiments involving wheel slip, delay, actuator nonlinearities, payload variation, and realistic sensing conditions.

## Supporting information

S1 File MATLAB source code and data used to reproduce the controller comparisons, trigger-parameter sensitivity and Pareto analyses, and paired Monte Carlo robustness results. (ZIP)
