# Supplementary material for: Event-triggered MPC-PID based trajectory tracking control for differential drive mobile robots
Source: PLoS One. 2026 Jul 29;21(7):e0354699. doi: 10.1371/journal.pone.0354699 (PMC13419250; doi:10.1371/journal.pone.0354699)
Supplement: Attachment — Submitted filename: comments.pdf [file pone.0354699.s003.pdf]

The manuscript proposes an event-triggered MPC-PID hierarchical control framework for trajectory tracking of differential-drive mobile robots. The separation among the MPC setpoint, actuator command, and actual actuator velocity is clearly explained, and the ablation study provides useful evidence regarding the role of the inner-loop PID servo. Nevertheless, several issues should be addressed before the manuscript can be considered for publication.

1. The proposed method appears to combine two established ideas: event-triggered MPC and hierarchical MPC-PID control. Although the manuscript explains their integration and provides a qualitative comparison in Table 1, it remains unclear whether the contribution represents a fundamentally new control formulation or mainly an engineering combination of existing components. The authors should more explicitly identify the mathematical or algorithmic differences from the closest event-triggered MPC and MPC-PID methods. A direct numerical comparison with at least one representative event-triggered MPC method under the same robot model, constraints, actuator dynamics, and computational platform would substantially strengthen the novelty claim.

2. Assumption 2 presumes that the nominal periodic MPC is locally stabilizing, while Assumption 3 directly assumes recursive feasibility. Consequently, an important part of the desired closed-loop properties is assumed rather than derived from the proposed optimization problem. The manuscript should provide verifiable sufficient conditions for these assumptions, for example through an appropriate terminal cost, terminal set, local invariant region, or explicit constraints on the prediction horizon and weighting matrices. It would also be valuable to derive an explicit ultimate tracking-error bound in terms of the maximum holding time, actuator mismatch bound, linearization residual, and triggering threshold. Otherwise, the theoretical claims should be moderated and described as a local boundedness characterization rather than a complete stability guarantee.

3. The event-triggering rule contains several normalization constants, weighting coefficients, a global threshold, and minimum and maximum inter-event times. Moreover, some parameters are modified between the double lane-change and figure-eight cases. The current results therefore do not establish whether the reported performance is robust or highly dependent on manual tuning. The authors should explain how the equivalent inner-loop time constant, reference-variation bound, and allowable holding error used in the proposed tuning guidelines are calculated. A sensitivity or Pareto analysis with respect to the triggering threshold and maximum holding time should also be provided, showing the trade-off among tracking error, control smoothness, and MPC call rate.

4. The current evaluation is limited to MATLAB/Simulink simulations with an ideal kinematic plant and prescribed first-order actuator dynamics. Important effects encountered by differential-drive robots, including wheel slip, sensor noise,

communication or computation delay, actuator dead zones, parameter uncertainty, payload variation, and external disturbances, are not examined. Physical robot experiments would be highly desirable. If hardware experiments are not currently possible, the authors should at least conduct Monte Carlo simulations with randomized initial conditions, actuator parameters, disturbances, measurement noise, and model mismatch. Solver failures, constraint violations, and worst-case tracking errors should also be reported.

5. Reducing the number of QP calls does not necessarily imply a proportional reduction in total real-time computational burden. The authors should report the processor, memory, MATLAB version, solver configuration, QP dimensions, initialization or warm-start strategy, and the computational overhead of the triggering and PID modules. In addition to the average QP time, the maximum and preferably the 95th-percentile execution times should be given and compared with the 20-ms sampling interval. A unified quantitative table should report RMSE or IAE, maximum position and heading errors, control variation, constraint violations, MPC call rate, and total computation time for all six controllers. Repeated trials and variability statistics would make the comparative conclusions considerably more reliable.
