# Supplementary material for: Event-triggered MPC-PID based trajectory tracking control for differential drive mobile robots
Source: PLoS One. 2026 Jul 29;21(7):e0354699. doi: 10.1371/journal.pone.0354699 (PMC13419250; doi:10.1371/journal.pone.0354699)
Supplement: Attachment — Submitted filename: Response to Reviewers.docx [file pone.0354699.s004.docx]

**Response to Reviewers**

**Manuscript ID: PONE-D-26-22794**

*Event-triggered MPC-PID based trajectory tracking control for differential drive mobile robots*

**Dear Academic Editor and Reviewers,**

The authors would like to express their sincere appreciation to the Academic Editor and the reviewers for their careful evaluation, valuable comments, and detailed suggestions. These comments have been very helpful for improving the motivation, technical formulation, presentation, and clarity of the manuscript.

According to the comments, we have revised the manuscript carefully. The main changes include clarifying the novelty and research question, enriching the literature review, adding a comparative related-work table, revising the modeling and controller design, adding anti-windup and actuator-mismatch analysis, strengthening the practical stability discussion, adding ablation studies, improving the figures, and revising the English writing and format. The corresponding changes in the revised manuscript are highlighted in blue in the marked-up version.

A clean revised manuscript and a marked-up manuscript have been prepared. Unless otherwise stated, line numbers in this response refer to the clean revised manuscript.

**Summary of major revisions**

• The Abstract and Conclusions were revised to state the novelty and contribution more clearly.

• The Introduction was revised to include a clearer research question, updated literature, practical application context, and a new comparison table distinguishing event-triggered, hierarchical, actuator/servo, and stability/robustness features.

• The modeling section now distinguishes the MPC setpoint, saturated actuator command, and actual actuator velocity, and the actuator mismatch is decomposed into holding-, lag-, and saturation-induced components.

• The controller design now includes an anti-windup PID velocity servo, a clarified first-order actuator model, and a discussion of the matching between event-triggered holding time and inner-loop response time.

• The stability analysis was revised to discuss practical stability / ultimate boundedness under bounded holding time and bounded actuator mismatch.

• The Results and Discussion section now includes ablation studies using ET-MPC-same and ET-MPC-retuned, together with revised/new figures and Table 4 to quantify tracking performance and computational cost.

• References, English writing, figure quality, and formatting were checked and revised according to the journal requirements.

**Responses to Journal Requirements and Academic Editor Comments**

**Journal Requirement 1: PLOS ONE style and file naming**

**Comment**
Please ensure that your manuscript meets PLOS ONE style requirements, including those for file naming.

**Response:** Thank you for the reminder. We have checked the manuscript against the PLOS ONE LaTeX template and revised the section headings, captions, line numbering, bibliography style, and reference formatting. The clean manuscript is prepared as the unmarked version, and the marked-up manuscript highlights the changes made to the original version. We will also follow the journal file-naming requirements during submission.

**Location in the revised manuscript:** Whole manuscript; references section; figure and table captions throughout.

**Journal Requirement 2: Code sharing**

**Comment**
Please ensure that author-generated code is shared in accordance with the journal policy.

**Response:** Thank you for pointing this out. We have prepared the author-generated MATLAB/Simulink scripts used for the simulations and for generating the figures and tables. These files include the simulation setup, controller implementation, ablation scripts, and plotting/statistical scripts needed to reproduce the reported results, and will be uploaded as Supporting Information with the revised submission.

**Location in the revised manuscript:** Data Availability Statement / Supporting Information files to be uploaded with the revision.

**Journal Requirement 3: Minimal data set**

**Comment**
Please confirm whether the submission contains all raw data required to replicate the results, including values behind figures and summary statistics.

**Response:** Thank you for this important requirement. We have prepared the minimal dataset needed to reproduce the findings reported in the manuscript. The dataset includes the numerical values used to generate Figs 3-11, the simulation and controller parameters summarized in Table 3, and the computational-cost/statistical values reported in Table 4. No external datasets were used in this study. The dataset will be uploaded as Supporting Information with the revised submission.

**Location in the revised manuscript:** Data Availability Statement / Supporting Information files to be uploaded with the revision.

**Journal Requirement 4: Separate figure files**

**Comment**
Please remove figures from within the manuscript file and upload individual TIFF/EPS figure files separately.

**Response:** Thank you for the instruction. We have prepared individual EPS figure files for Figs 1-11. In the final submission package, the figures will be uploaded separately according to the PLOS ONE requirements, while the manuscript will retain the corresponding captions and in-text figure references.

**Location in the revised manuscript:** Figs 1-11; final submission package.

**Journal Requirement 5: Suggested literature**

**Comment**
If reviewer comments include a recommendation to cite specific previously published works, please review and evaluate these publications.

**Response:** We appreciate this instruction. We reviewed the relevant event-triggered PID, predictive control, and integrated guidance-control studies suggested by the reviewers. Relevant works were added and discussed in the Introduction, including event-triggered adaptive PID fault-tolerant control for underactuated ASVs and FTILOS-based path following for underactuated USVs. We also added recent literature from 2020-2026 on MPC, event-triggered MPC, hierarchical MPC-PID control, and mobile robot trajectory tracking.

**Location in the revised manuscript:** Introduction, lines 42-95; References.

**Academic Editor: Motivation, originality, formulation, and writing issues**

**Comment**
The paper is not well motivated and its originality is unclear because there are many existing studies on ET-based PID control and applications. There are also formulation and writing issues that need refinement.

**Response:** Thank you for your careful evaluation and helpful summary. We are sorry that the motivation and originality were not sufficiently clear in the previous version. We have revised the manuscript carefully to address these issues. The revised Introduction now explicitly states the research gap and clarifies that the hierarchical MPC-PID architecture itself is not claimed as the sole novelty. The intended contribution is the integration of an event-triggered MPC outer loop with a fixed-period PID velocity servo while explicitly considering actuator dynamics, input saturation, setpoint holding, and bounded triggering frequency. A new Table 1 was added to compare related methods in terms of event-triggering, hierarchy, actuator/servo treatment, and stability/robustness. We also revised the modeling formulation, clarified the input signal chain, strengthened the practical stability analysis, added ablation studies, and reviewed the English throughout.

**Location in the revised manuscript:** Introduction, lines 85-131; modeling, lines 152-212 and 239-298; controller and stability analysis, lines 308-498; Results and Discussion, lines 499-625.

**Responses to Reviewer #1**

**Reviewer #1, Comment 1: Abstract and conclusion should highlight novelty**

**Comment**
In the abstract and conclusion, the contribution of this paper is not well presented. Highlight the novelty of the paper.

**Response:** Thank you for this valuable comment. We are sorry that the novelty and contribution were not sufficiently highlighted in the original Abstract and Conclusion. We revised both parts to make the contribution clearer. The revised Abstract now states that the novelty lies in applying event-triggered optimization to the MPC outer loop while using a fixed-period PID velocity servo to compensate for actuator lag, input saturation, and setpoint holding effects. The revised Conclusions summarize the contribution in terms of the event-triggered MPC outer loop, fixed-period PID velocity servo, explicit treatment of setpoint holding/actuator lag/input saturation, anti-windup compensation, and ablation validation.

**Location in the revised manuscript:** Abstract, page 1; Conclusions and Perspectives, lines 626-644.

**Reviewer #1, Comment 2: Weak Introduction, research question, aim, and contribution**

**Comment**
The introduction is weak and should include the research question, the aim of the paper, and the contribution.

**Response:** Thank you for your constructive suggestion. We have revised the Introduction to better explain the motivation, aim, research question, and contribution of the study. The revised Introduction now begins with practical trajectory-tracking challenges for differential-drive robots, then discusses the computational redundancy of periodic MPC, the practical role of hierarchical MPC-PID structures, and the limitations of existing ET-MPC studies. The research question is now explicitly stated as whether the online optimization frequency of constrained MPC can be reduced without sacrificing practical trajectory-tracking performance when low-level actuator response is considered. The contribution list was also rewritten to clearly present the aim and main contributions of the paper.

**Location in the revised manuscript:** Introduction, lines 1-15, 16-41, 42-84, 96-131.

**Reviewer #1, Comment 3: Novelty compared with previous work and related-work criticism**

**Comment**
Many researches work on this idea. What is really the novelty as compared to other studies? What is the new and the difference between the previous works and present work? The explanation of the related work needs to be criticized and improved in general.

**Response:** Thank you for pointing out this important issue. We agree that the difference from previous work needed to be explained more clearly. We have added a more critical discussion of representative periodic MPC, MPC-PID, ET-MPC, ET-PID, and USV/ASV control studies. In addition, a new Table 1 was added to compare existing methods according to whether they are event-triggered, whether they are hierarchical, whether they consider actuator dynamics/servo effects, and whether stability/robustness is considered. The revised text clarifies that the proposed method is not merely a generic hierarchical MPC-PID design. Instead, it focuses on an event-triggered outer-loop MPC combined with a fixed-period inner-loop PID velocity servo, while explicitly considering actuator mismatch and validating the roles of triggering and inner-loop servoing through ablation studies.

**Location in the revised manuscript:** Introduction, lines 28-95; Table 1; contribution statement, lines 96-131.

**Reviewer #1, Comment 4: Improve latest literature from 2020-2026 and applications**

**Comment**
Improve the quality of literature along with the latest literature. What about last updating in this topic and new references from 2020-2026? The survey of existing literature is not sufficient. It would useful to include in the Introduction of the paper some discussion on other possible real applications of the obtained results.

**Response:** Thank you for your helpful suggestion. We updated and enriched the literature review with recent studies from 2020-2026. The revised Introduction now discusses recent works on geometric MPC for wheeled mobile robots, Lyapunov-constrained MPC, nonlinear disturbance-observer predictive control, edge-assisted MPC for AGV/AMR navigation, MPC-PID double-loop tracking for intelligent sweeping vehicles, recent event-triggered control surveys, ET-MPC methods, event-triggered predictive control for unmanned/autonomous vehicles, disturbance event-triggered predictive tracking for mobile robots, and ET-PID/USV/ASV studies. We also added application contexts including environmental inspection, warehousing logistics, service robotics, intelligent manufacturing, AGV/AMR navigation, and intelligent sweeping vehicles.

**Location in the revised manuscript:** Introduction, lines 1-84; References 1-26.

**Reviewer #1, Comment 5: Figure quality and visibility**

**Comment**
Figures are not clear. Clear diagrams and figures are required for readers to have clear images. Improve the quality of figures for better visibility. It is blur that should be adjusted.

**Response:** Thank you for your comment. We have revised and regenerated the figures to improve clarity and readability. The overall architecture figure was redrawn to show the event-triggering mechanism, outer-loop MPC, memory/hold unit, inner-loop PID velocity servo, saturation, actuator dynamics, robot kinematics, and feedback path more clearly. The simulation figures were also updated or newly added to show trajectory/ablation comparison, inter-event intervals, tracking errors, control input, execution-chain consistency, and summary metrics. These revised figures are provided as individual EPS files for submission.

**Location in the revised manuscript:** Fig 2; Figs 3-11; Results and Discussion, lines 518-625.

**Reviewer #1, Comment 6: Weak conclusion and future work**

**Comment**
Weak conclusion and the future work were meaningless in this article. Conclusion should be more specific with improvement writing quality. A suggestion for future work should be added in the conclusion section.

**Response:** Thank you for this valuable suggestion. We have revised the Conclusions and Perspectives section to make it more specific. The revised conclusion summarizes the main technical contribution, the distinction among the MPC setpoint, actuator command, and actual actuator velocity, the practical stability interpretation, the anti-windup mechanism, and the ablation study findings. The future work now includes physical robot experiments under sensor noise, communication delay, wheel slip, and actuator dead zones, as well as adaptive event-triggering and learning-based parameter tuning.

**Location in the revised manuscript:** Conclusions and Perspectives, lines 626-644.

**Reviewer #1, Comment 7: References, formatting, and English writing**

**Comment**
Rewrite the references according to journal template. Please strictly follow the instructions to the format specified in the journal template for preparing the paper. The format and English writing of this paper should be improved. The paper needs language revision.

**Response:** Thank you for your careful reminder. We revised the references using the PLOS bibliography style and checked the formatting according to the PLOS ONE template. We also reviewed the manuscript for grammar, clarity, terminology consistency, and formatting. Method names such as ET-MPC-PID, ET-MPC-same, and ET-MPC-retuned were standardized, and unclear expressions were rewritten throughout the manuscript.

**Location in the revised manuscript:** Whole manuscript; References section.

**Reviewer #1, Comment 8: Data availability**

**Comment**
Reviewer #1 indicated that the data underlying the findings were not fully available.

**Response:** Thank you for pointing this out. We have prepared the minimal dataset and author-generated code as Supporting Information. The data files include the numerical values required to reproduce the reported figures, summary metrics, and computational-cost results. The code files include the MATLAB/Simulink scripts used to generate the reported results. No external datasets were used in this study.

**Location in the revised manuscript:** Data Availability Statement / Supporting Information files to be uploaded with the revision.

**Responses to Reviewer #2**

**Reviewer #2, Comment 1: Introduction should clearly distinguish existing ET-MPC and the proposed novelty**

**Comment**
In the introduction of the paper, the general operation of event-triggered MPC is listed, and references that apply event-triggering to trajectory tracking are mentioned. However, the author should conduct a detailed analysis of the essential differences from existing methods. The novelty should be stated as integrating MPC with PID in a hierarchical manner and using event-triggering in the outer loop, while the hierarchical structure itself is not novel. A tabular comparison is recommended.

**Response:** Thank you very much for this insightful comment. We agree with the reviewer and have revised the Introduction accordingly. The revised manuscript now explicitly states that the hierarchical MPC-PID architecture itself is not claimed as the sole novelty. Instead, the intended contribution is integrating an event-triggered outer-loop MPC with a fixed-period inner-loop PID velocity servo and explicitly evaluating actuator dynamics, input saturation, setpoint holding, and bounded triggering frequency. A new Table 1 was added to compare related methods with respect to event-triggering, hierarchy, actuator/servo handling, and stability/robustness. The surrounding text explains how existing periodic MPC, MPC-PID, ET-MPC, ET-PID, and USV/ASV studies differ from the present framework.

**Location in the revised manuscript:** Introduction, lines 56-110; Table 1; contribution statement, lines 111-131.

**Reviewer #2, Comment 2: Matching between event-triggered holding time and inner-loop PID response**

**Comment**
The inner-loop PID may not fully compensate for tracking lag during long event-triggered holding periods. The impact of this coupling and the quantitative relationship between inner-loop bandwidth and outer-loop trigger interval should be discussed.

**Response:** Thank you for your valuable comment. We have added a discussion on the interaction between the event-triggered holding time and the inner-loop response capability. Specifically, we introduced a minimum inter-event time and maximum holding time, and added a practical tuning guideline involving the equivalent inner-loop closed-loop response time constant, the desired decay ratio, the upper bound on reference variation rate, and allowable holding-induced error. This clarifies that $T_{min}$ould avoid unnecessary re-optimization before the inner loop responds sufficiently, while $T_{max}$mits the maximum duration of holding a previous MPC setpoint. In addition, the stability analysis and ablation study now explicitly explain the accuracy-computation trade-off and show how ET-MPC-same, ET-MPC-retuned, and ET-MPC-PID differ under different holding/update behaviors.

**Location in the revised manuscript:** Event-triggering mechanism, lines 404-426; Stability analysis and ablation explanation, lines 427-498; Results and Discussion, lines 499-625.

**Reviewer #2, Comment 3: Saturation and actuator mismatch should be explicitly handled and quantified**

**Comment**
Although input and input increment constraints are added to the optimization problem, the actual actuator still has saturation and first-order inertia. The actual input may deviate from the ideal input assumed in the MPC prediction. This deviation should be explicitly considered, with anti-saturation compensation or a clearer inner-loop design.

**Response:** Thank you for this important suggestion. We revised both the modeling and controller design to address this issue. The revised manuscript now distinguishes three velocity signals: the MPC setpoint, the saturated actuator command, and the actual actuator velocity. The actuator command is subject to component-wise saturation, and the actual velocity follows first-order actuator dynamics. We defined the actuator mismatch and decomposed it into holding-, lag-, and saturation-induced terms. In the inner loop, an anti-windup correction was added to suppress excessive integral accumulation under saturation. The stability analysis was also revised to express the closed-loop error dynamics as a nominal stabilizing MPC loop perturbed by bounded event-holding, actuator-lag, and saturation-induced mismatch, leading to an ultimate boundedness result. Fig 10 was added to verify the consistency among setpoint, command, and actual actuator velocity.

**Location in the revised manuscript:** Modeling, lines 152-212 and 239-257; inner-loop PID and anti-windup, lines 357-379; stability analysis, lines 427-492; Fig 10 and related text in Results and Discussion.

**Reviewer #2, Comment 4: Include ET-based PID and related ASV/USV studies**

**Comment**
Existing ET-based PID control methods and their applications, such as event-triggered adaptive PID fault-tolerant control of underactuated ASVs under saturation constraint and FTILOS-based path following for underactuated USVs with obstacle avoidance, should be considered to highlight the contribution.

**Response:** Thank you for recommending these relevant studies. We have added and discussed these works in the revised Introduction. The discussion explains that such studies are relevant because they address event-triggering, saturation, fault tolerance, obstacle avoidance, and integrated guidance-control. We also clarified that their control objectives, plant models, and design structures differ from the present differential-drive mobile robot problem and from the proposed outer-loop ET-MPC plus inner-loop PID velocity-servo framework. These works were also included in Table 1 to make the comparison more explicit.

**Location in the revised manuscript:** Introduction, lines 70-84; Table 1; References 25-26.

**Reviewer #2, Comment 5: Grammar and unclear expressions**

**Comment**
The manuscript contains numerous grammatical errors and unclear expressions. The author should carefully review it and eliminate such unnecessary mistakes.

**Response:** Thank you for your careful reading. We have revised the whole manuscript for clarity, grammar, and consistency. Several sections were rewritten or restructured, including the Abstract, Introduction, modeling formulation, controller design, stability analysis, Results and Discussion, and Conclusions. We also standardized terminology and notation throughout the manuscript. The major changes are highlighted in the marked-up manuscript.

**Location in the revised manuscript:** Whole manuscript; major revisions are highlighted in the marked-up manuscript.

**Closing statement**

The authors sincerely thank the Academic Editor and both reviewers again for their valuable comments and suggestions. We hope that the revisions have addressed the concerns and have improved the motivation, novelty statement, technical formulation, stability discussion, experimental validation, figure quality, and writing clarity of the manuscript.

**Sincerely,**

Maike Wang, Gang Zhang, and Duansong Wang
