# Supplementary material for: Event-triggered MPC-PID based trajectory tracking control for differential drive mobile robots
Source: PLoS One. 2026 Jul 29;21(7):e0354699. doi: 10.1371/journal.pone.0354699 (PMC13419250; doi:10.1371/journal.pone.0354699)
Supplement: Attachment — Submitted filename: Response_to_Reviewers_auresp_2.docx [file pone.0354699.s005.docx]

**Response to Reviewers**

**Manuscript ID: PONE-D-26-22794R1**

*Event-triggered MPC-PID based trajectory tracking control for differential drive mobile robots*

**Dear Academic Editor and Reviewers,**

The authors sincerely thank the Academic Editor and the reviewers for their careful evaluation and constructive comments. We are grateful that Reviewer #1 considered the previous concerns adequately addressed. We particularly appreciate Reviewer #3 for identifying the need to sharpen the novelty statement, moderate the theoretical claims, strengthen the triggering-parameter analysis, expand the uncertainty evaluation, and report a fuller computational profile.

In response, we have revised the manuscript extensively. The revised work now clarifies that the contribution is an event-triggered outer MPC combined with a fixed-period PID velocity servo and an explicit treatment of holding-induced and actuator-side discrepancies, rather than a claim of a wholly new standalone MPC-PID architecture. We added a same-platform external VH-ET-MPC reference, internal ablation controllers, a local practical-boundedness characterization, common nominal triggering settings supported by sensitivity and Pareto analyses, paired 100-trial Monte Carlo studies, and detailed runtime diagnostics. The revised manuscript also reports the limitations of the present simulation-based assessment.

A clean revised manuscript and a marked-up manuscript have been prepared. Unless otherwise stated, line numbers in this response refer to the clean revised manuscript.

**Summary of Major Revisions**

• The Abstract, Introduction, and Conclusions were revised to state the scope, novelty, theoretical boundary, and empirical evidence more precisely.

• A same-platform variable-horizon ET-MPC reference and two internal ablations (ET-MPC-same and ET-MPC-retuned) were incorporated to distinguish the effects of event updating, update density, and fixed-period velocity compensation.

• The model now explicitly separates the MPC setpoint, saturated actuator command, actual actuator velocity, holding-induced decision discrepancy, and actuator-side execution mismatch.

• Theoretical claims were moderated to a local practical-boundedness characterization. The explicit perturbation bound now contains the trigger threshold, maximum holding time, actuator-related terms, and local linearization residual.

• All nominal trigger parameters, normalization scales, weights, VH-ET-MPC settings, implementation details, and computing specifications were reported. A two-parameter sensitivity and Pareto study supports the common nominal configuration.

• Paired 100-trial Monte Carlo studies and runtime diagnostics were added, including feasibility, terminal success, QP failures, constraint violations, worst maximum position errors, average/P95/maximum QP times, and trigger/PID overheads.

**Response to the Academic Editor**

**Editorial decision and submission requirements:** We thank the Academic Editor for inviting a revision. The revised submission includes a response letter, a marked-up manuscript, and a clean manuscript, as requested. Individual figure files and the supporting materials containing the author-generated simulation scripts and underlying numerical results will be organized and supplied with the revised submission in accordance with the journal requirements.

**Reviewer #1:** We sincerely thank Reviewer #1 for confirming that the previous comments have been addressed and that the manuscript is acceptable without further changes.

**Responses to Reviewer #3**

**Reviewer #3, Comment 1: Novelty and direct ET-MPC comparison**

**Comment:** *The contribution appears to combine established event-triggered MPC and hierarchical MPC-PID ideas. The manuscript should identify the mathematical or algorithmic differences from the closest methods and provide a direct numerical comparison with a representative ET-MPC method under the same robot model, constraints, actuator dynamics, and computational platform.*

**Response:** Thank you for this important comment and for recognizing the value of the signal separation and ablation design. We agree that the previous version did not sufficiently distinguish the contribution from a generic combination of ET-MPC and hierarchical MPC-PID. We have therefore revised the novelty claim and the comparison protocol. The Introduction now states explicitly that the hierarchical MPC-PID architecture is not claimed as a stand-alone novelty. The contribution is the event-triggered constrained MPC outer loop combined with a fixed-period PID-form velocity servo, together with an explicit distinction between holding-induced decision discrepancy and actuator-side execution mismatch. Table 1 was expanded to compare representative periodic MPC, MPC-PID, ET-MPC, and related methods. We also implemented an external variable-horizon ET-MPC reference (VH-ET-MPC) following the prediction-mismatch, stored-sequence, and contracting-horizon principles of Ref. [24]. This external reference uses the same differential-drive model, sampling period, reference trajectories, QP solver, input/input-rate constraints, and actuator model as the proposed method. In addition, ET-MPC-same removes only the fixed-period PID servo under the same nominal trigger configuration, whereas ET-MPC-retuned deliberately increases the update density without the PID servo. These comparisons allow the effects of the event policy, inner-loop compensation, and update frequency to be distinguished without claiming a fundamentally new control theory.

**Location in the revised manuscript:** Introduction, lines 65–144 and Table 1; signal-chain and discrepancy definitions, lines 193–220; external VH-ET-MPC reference, lines 453–480; ablation definition, lines 554–562; same-platform simulation protocol and results, lines 571–625.

**Reviewer #3, Comment 2: Theoretical assumptions and practical boundedness**

**Comment:** *The nominal periodic MPC stability and recursive feasibility appear to be assumed rather than derived. The manuscript should provide sufficient conditions or otherwise moderate the theory to a local boundedness characterization. The ultimate tracking-error bound should show the effects of maximum holding time, actuator mismatch, linearization residual, and trigger threshold.*

**Response:** Thank you for this rigorous comment. We agree that the original presentation could be interpreted as claiming stronger closed-loop guarantees than were justified by the finite-horizon QP formulation. Rather than adding terminal ingredients that are not part of the implemented controller, we have moderated the theoretical claim. The revised manuscript now presents the analysis explicitly as a local practical-boundedness characterization, not as a global asymptotic-stability or complete recursive-feasibility proof. The local operating region, local stabilizing behavior of the nominal periodic MPC, local feasibility, bounded actuator-side mismatch, and bounded holding perturbation are stated transparently as assumptions. The aggregate perturbation is decomposed into holding-, lag-, saturation-, and local-linearization-residual terms. Equation (69) gives an explicit bound that depends on the trigger threshold, maximum holding time, actuator-related bounds, and linearization residual; Equation (82) then gives the resulting local ultimate bound. The text also states the scope and limitation of this result directly.

**Location in the revised manuscript:** Scope of the local analysis, lines 481–491; local assumptions, lines 492–524; perturbation decomposition and explicit bound, lines 525–534; Lyapunov-based local practical bound, lines 535–553; limitation statement, lines 563–569.

**Reviewer #3, Comment 3: Trigger-parameter transparency and robustness**

**Comment:** *The event rule contains several normalization constants, weights, thresholds, and holding times, and some parameters were modified between trajectories. The manuscript should explain how the equivalent inner-loop time constant, reference-variation bound, and allowable holding error are calculated, and should provide sensitivity or Pareto analysis for the trigger threshold and maximum holding time.*

**Response:** Thank you for highlighting the need for a transparent parameter-selection procedure. We have clarified the role and numerical values of the trigger quantities in Table 3, including the normalization scales, weighting matrix, common nominal threshold, and minimum/maximum holding times. The final nominal ET-MPC-same and ET-MPC-PID comparisons now use the same configuration in both trajectories: Jthr = 0.90, Tmin = 0.08 s, and Tmax = 0.12 s; curvature triggering is disabled. The retuned ablation is intentionally reported separately because it is designed to test whether increasing the update frequency alone can replace fixed-period inner-loop compensation. We also operationally define τcl as the larger 95% settling time of the two closed-loop PID-actuator velocity channels, calculate Lr from the maximum sampled reference-variation rate, and define ehold-bar as an engineering tracking-error budget for interpreting the holding interval. Finally, a 25-point two-parameter sensitivity analysis and a Pareto plot quantify the trade-off among tracking accuracy, maximum position error, control variation, and MPC call rate. These analyses support the common nominal configuration as a shared compromise rather than a separately optimized setting for each trajectory.

**Location in the revised manuscript:** Trigger definition and normalizations, lines 396–420; holding-time guidelines and operational definitions, lines 422–451; Table 3; common nominal setting and ablation protocol, lines 586–607; sensitivity and Pareto analyses, lines 680–696 and Figs. 10–11.

**Reviewer #3, Comment 4: Uncertainty evaluation and robustness reporting**

**Comment:** *The evaluation is limited to simulations. If hardware experiments are not available, Monte Carlo simulations with randomized initial conditions, actuator parameters, disturbances, measurement noise, and model mismatch should be added. Solver failures, constraint violations, and worst-case tracking errors should also be reported.*

**Response:** Thank you for this constructive recommendation. Physical robot experiments remain an important next step, but they are outside the scope of the present revision. To strengthen the numerical evidence, we added paired 100-trial Monte Carlo studies for each trajectory. Within every trial, all compared controllers receive the same initial-state perturbation, actuator realization, measurement-noise sequence, and disturbance realization. The study includes randomized initial position and heading offsets, perturbed actuator time constants and gains with clipping bounds, Gaussian position and heading measurement noise, and additive equivalent linear- and angular-velocity disturbances. The revised manuscript reports feasibility rate, terminal-success rate, position-RMSE mean, standard deviation and P95, worst maximum position error, MPC call rate, QP failures, and constraint-violation steps. The text also makes clear that this is an empirical robustness evaluation under sampled execution uncertainty, not a probabilistic extension of the deterministic local analysis. For transparency, the proposed controller records one QP failure in the 100 figure-eight trials and no constraint-violation time step; all other reported Monte Carlo diagnostics are given in Table 6. The limitations and the need for physical validation under wheel slip, delay, actuator nonlinearities, payload variation, and realistic sensing are stated in the conclusion.

**Location in the revised manuscript:** Connection between deterministic analysis and empirical robustness, lines 563–569; Monte Carlo design and uncertainty settings, lines 698–711; Monte Carlo outcomes and worst-case error reporting, lines 712–722 and Table 6; limitations and future work, lines 724–754.

**Reviewer #3, Comment 5: Computational burden and reproducibility**

**Comment:** *Reducing QP calls does not alone establish a proportional real-time computational reduction. The processor, memory, MATLAB version, solver configuration, QP dimensions, warm-start strategy, trigger/PID overhead, average/P95/maximum execution times, constraint violations, call rate, total computation time, and repeated-trial variability should be reported.*

**Response:** Thank you for this detailed recommendation. We substantially expanded the implementation and computational reporting. Table 3 now specifies the computing environment (Intel Core Ultra 5 225H CPU, 32 GB RAM, MATLAB R2024b under Windows), QP solver (MATLAB quadprog with the interior-point-convex algorithm), the absence of warm start, and the QP dimensions (61 decision variables, 120 cumulative-input inequalities, and box constraints). Table 4 reports nominal tracking performance, maximum position error, heading RMSE, control variation, QP call rate, solver failures, and constraint-violation information. Table 5 separately reports QP calls, call rate, mean/P95/maximum QP time, total QP time, and measured trigger/PID overhead, preserving readability while providing the full computational profile. We also compare the timing data with the 20-ms sampling interval and state explicitly that the MATLAB results demonstrate reduced average optimization burden but do not constitute a hard real-time guarantee on an embedded platform. The paired Monte Carlo study in Table 6 provides repeated-trial variability statistics and diagnostic outcomes under sampled uncertainty.

**Location in the revised manuscript:** Computing environment and implementation settings, Table 3; nominal tracking and diagnostic metrics, lines 661–669 and Table 4; timing analysis and real-time limitation, lines 670–679 and Table 5; repeated-trial statistics, lines 698–722 and Table 6.

**Closing Statement**

The authors sincerely thank the Academic Editor and the reviewers again for their valuable comments and suggestions. We believe that the revised manuscript now provides a clearer novelty statement, more transparent theoretical scope, stronger same-platform comparisons, more complete parameter and runtime reporting, and broader empirical robustness evidence. We hope that the revisions adequately address the concerns and that the manuscript is now suitable for publication in PLOS ONE.

**Sincerely,**

Maike Wang, Gang Zhang, and Duansong Wang
